# Imaging nanoscale nuclear structures with expansion microscopy

**DOI:** 10.1242/jcs.259009

**Published:** 2022-07-19

**Authors:** Emma L. Faulkner, Jeremy A. Pike, Ruth M. Densham, Evelyn Garlick, Steven G. Thomas, Robert K. Neely, Joanna R. Morris

**Affiliations:** 1 School of Chemistry, University of Birmingham, Edgbaston, Birmingham, B15 2TT, UK; 2 Institute of Cardiovascular Sciences, College of Medical and Dental Sciences, University of Birmingham, Birmingham, B15 2TT, UK; 3 The Centre of Membrane Proteins and Receptors (COMPARE), University of Birmingham and University of Nottingham, Birmingham, B15 2TT, UK; 4 Birmingham Centre for Genome Biology, University of Birmingham, Birmingham, B15 2TT, UK; 5 Institute of Cancer and Genomic Sciences, University of Birmingham, Birmingham, B15 2TT, UK

**Keywords:** Expansion microscopy, Nanoscale, 53BP1, BRCA1, RAD51, DNA damage

## Abstract

Commonly applied super-resolution light microscopies have provided insight into subcellular processes at the nanoscale. However, imaging depth, speed, throughput and cost remain significant challenges, limiting the numbers of three-dimensional (3D) nanoscale processes that can be investigated and the number of laboratories able to undertake such analysis. Expansion microscopy (ExM) solves many of these limitations, but its application to imaging nuclear processes has been constrained by concerns of unequal nuclear expansion. Here, we demonstrate the conditions for isotropic expansion of the nucleus at a resolution equal to or better than 120–130 nm (pre-expansion). Using the DNA damage response proteins BRCA1, 53BP1 (also known as TP53BP1) and RAD51 as exemplars, we quantitatively describe the 3D nanoscale organisation of over 50,000 DNA damage response structures. We demonstrate the ability to assess chromatin-regulated events and show the simultaneous assessment of four elements. This study thus demonstrates how ExM can contribute to the investigation of nanoscale nuclear processes.

## Abstract (precis)

**Summary:** Expansion microscopy provides quantitative insight into the impact of chromatin modifiers on spatiotemporal organisation of the DNA repair proteins BRCA1, 53BP1 and RAD51 at a resolution of 65–70 nm.

## INTRODUCTION

Major processes central to life occur within eukaryotic nuclei such that high-resolution imaging of nuclear structures is critical to improving our understanding of DNA replication, DNA repair, gene regulation and transcription. The application of fluorescence microscopy has provided insight into the organisation and regulation of many of these processes, and studies applying super-resolution microscopy (SRM) have allowed investigation of the spatial organisation of proteins within subcompartments with nanoscale resolution.

A particular example is DNA damage signalling, where repair proteins are redistributed in a spatiotemporally regulated manner to form microscopically visible aggregates, known as ‘foci’, around damaged sites (Bekker-Jensen et al., 2006). Application of confocal microscopy and SRM techniques, such as stimulated emission depletion (STED) microscopy, stochastic optical reconstruction microscopy (STORM) and structured illumination microscopy (SIM), have contributed to a spatial map of repair signalling in which the time of arrival and departure of a protein, and its relative site of residence, directs DNA repair pathways (Chapman et al., 2012; Kakarougkas et al., 2013; Bekker-Jensen et al., 2006; Reindl et al., 2017; Whelan et al., 2018; Ochs et al., 2019; Schwarz et al., 2019).

However, established super-resolution techniques offer a compromised solution to imaging the three-dimensional (3D) spatial organisation of nanoscale protein arrangements, typically requiring a trade-off between resolution and throughput (Schermelleh et al., 2018). Our current super-resolution view of DNA damage signalling, for example, is based on analysis of hundreds of structures with relatively low resolution (e.g. SIM imaging with a lateral resolution of 100–130 nm; Chapman et al., 2012) or tens of structures with improved resolution (e.g. STED microscopy and single-molecule localization microscopy imaging with lateral resolutions of 30–80 nm and 20 nm, respectively; Reindl et al., 2017; Whelan et al., 2018; Ochs et al., 2019). Moreover, because established SRM technologies require expensive equipment and sophisticated analytical tools, the number of laboratories able to investigate nanoscale structural organisations with the requisite sub-diffraction-limit resolution is restricted.

Expansion microscopy (ExM) has the potential to overcome some of the problems posed by other SRM modalities. However, whilst ExM has been successfully applied to the analysis of cytoplasmic structures, concerns about differential nuclear expansion and controversy over how samples should be prepared has limited the use of ExM to investigate nanoscale structures in the nucleus (Chozinski et al., 2016; Tillberg et al., 2016; Freifeld et al., 2017; Pesce et al., 2019; Pernal et al., 2020; Büttner et al., 2021).

Herein, we demonstrate conditions for isotropic expansion of the nucleus of human epithelial cells with minimal distortion. We investigate the nanoscale organisation of the DNA damage response proteins 53BP1 (also known as TP53BP1) and BRCA1, which have previously been assessed using established SRM techniques (Chapman et al., 2012; Reindl et al., 2017; Schwarz et al., 2019), and use manipulation of chromatin regulators underpinning 53BP1 localisation to demonstrate the ability of ExM to assess chromatin-regulated events. We assess thousands of nanoscale nuclear features, enabling unprecedented description of substructure heterogeneity, and illustrate 3D and four-colour analysis. These data demonstrate that ExM can be applied for the nanoscale analysis of nuclear structures at scale, offering an unparalleled insight into nuclear processes that is accessible to many laboratories.

## RESULTS

### Isotropic expansion of the nucleus

A key consideration in applying ExM is to avoid anisotropic expansion that can result in sample distortion (Geertsema and Ewers, 2016; Alon, et al., 2019). In ExM, specimens are labelled with conventional fluorescently labelled antibodies or proteins equipped with anchors that enable their incorporation into a dense and even polyelectrolyte gel meshwork formed throughout the sample. The sample is digested with proteases, and addition of water results in volumetric expansion of the gel, with the aim of retaining the relative spatial organisation of the labels (Chozinski et al., 2016; Chen et al., 2015; Tillberg et al., 2016). However, the presence of genomic DNA in the gel has been suggested to introduce distortions when the nucleus expands, adversely affecting isotropic expansion (Martínez et al., 2020; Pernal et al., 2020; Büttner et al., 2021).

We hypothesised that an approach in which nucleic acids are also anchored into the gel might both maintain the relative spatial organisation of nuclear structures, many of which relate to nucleic acid processing, and also promote isotropic expansion of the nucleus. To test this, we employed a nucleotide alkylating agent, conjugated to an acryolyl group through N-hydroxysuccinimide (NHS) ester chemistry, to form a compound termed ‘LabelX’ (Chen et al., 2016). This compound anchors polynucleotides into the gel network, but its impact on the nanoscale structure of the nucleus in ExM is unknown.

We first examined nuclei in U2OS cells that had been grown in the presence of the thymidine analogue 5-ethynyl-2′-deoxyuridine (EdU) to visualise the DNA via conjugation of a fluorescent azide (Salic and Mitchison, 2008). Following the application of the ExM protocol, we noted that nuclear areas were increased ∼16-fold (an expansion factor of 4 in one dimension) and that nuclear volumes were increased by ∼52-fold (an expansion factor of 3.7 in one dimension) (Fig. S1A–D). We found that nuclear expansion measurements corresponded to the fourfold macroscale expansion of the gel.

**Fig. 1.**
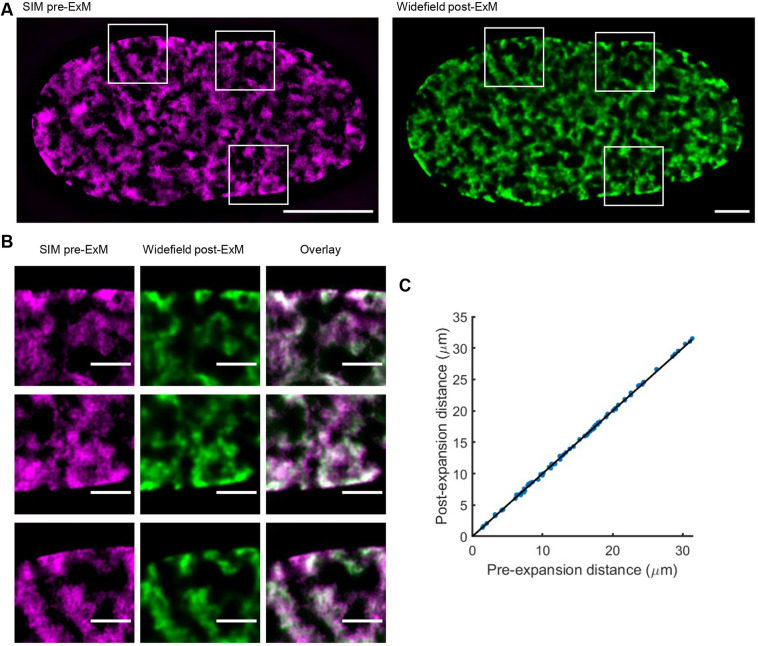
**Isotropic expansion of the nucleus.** U2OS cells were treated with EdU overnight prior to fixation. A Click-iT reaction was performed for detection of EdU, and images were acquired either pre-expansion (pre-ExM) or post-expansion (post-ExM). (A) Correlative imaging was performed by acquiring pre-ExM images on a structured illumination microscope in 3D-SIM mode and then acquiring images of the same nuclei post-ExM on a widefield microscope. Boxes indicate regions shown in B. Scale bars: 10 μm. (B) Features were selected from the registered pre- and post-ExM correlative images and compared. Overlays of these features pre-ExM and post-ExM are shown. Scale bars: 2 μm. Scale bars correspond to pre-expansion size. Images in A and B are representative of one experiment. (C) Distances between pairs of control points in the pre-ExM and post-ExM images. Data shown are 21 manually placed points in the nucleus shown, representative of one experiment. The MSE between pairwise control points was determined.

To assess the isotropy at the nanoscale, the same U2OS cell nuclei were imaged pre- and post-expansion, and features within these images were compared (Fig. 1A). Axial expansion of the sample changed the imaging depth of field. Nevertheless, we were able to confirm that the morphology of the nuclei was retained, with the nanoscale features identified pre-expansion readily observable post-expansion with no distortions evident (Fig. 1B). These data suggest that in the presence of the LabelX anchor, the nucleus expands isotropically on the micro- and nano-scale with distortions to the genomic architecture that are not observable at a resolution of 120–130 nm laterally. We further quantified the isotropy of expansion by measuring mean squared error (MSE) between points of interest in the pre- and post-expansion images (Fig. 1C). We further confirmed this was reproducible across biological replicates (data not shown).

### Nanoscale organisation of DNA damage signalling proteins

We next investigated the organisation of the pivotal DNA double-strand break repair regulator proteins BRCA1 and 53BP1 in S-phase U2OS cells following exposure to gamma irradiation (2 Gy). The relative spatial organisation of these proteins under such conditions has previously been characterised using confocal and super-resolution techniques (Chapman et al., 2012; Kakarougkas et al., 2013; Ochs et al., 2019; Schwarz et al., 2019).

**Fig. 2.**
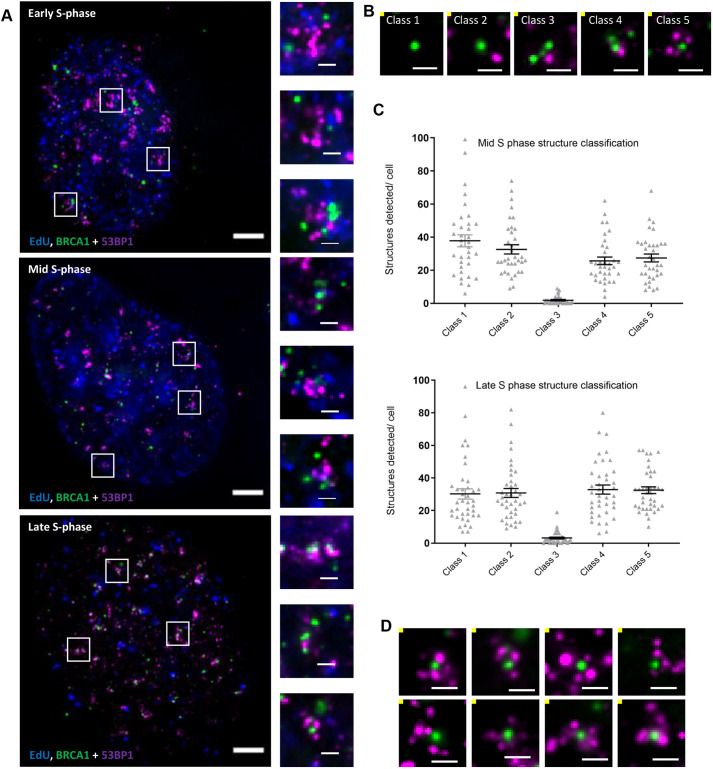
**Characterising nanoscale organisation of the DNA damage signalling proteins 53BP1 and BRCA1.** U2OS cells were treated with EdU (blue) and damaged with irradiation (2 Gy) before being allowed to recover for 1 h prior to fixation. Cells were immunostained for BRCA1 (green) and 53BP1 (magenta), then prepared using the ExM method. (A) Post-expansion images of nuclei classified as early, mid and late S-phase, as indicated. Boxes indicate regions shown in magnified views on the right. Scale bars: 10 μm (main images) and 2 μm (selected regions), equivalent to ∼2.5 μm and ∼500 nm pre-expansion, respectively. (B) Representative images of structure classes 1–5 containing BRCA1 spots (green) and 53BP1 spots (magenta). Scale bars: 2 μm (equivalent to 500 nm pre-expansion). Class 1 structures were comprised of only the core BRCA1 spot. Class 2 structures contained the core BRCA1 spot and a 53BP1 spot. Class 3 structures contained multiple BRCA1 spots and one 53BP1 spot. Class 4 structures incorporated multiple 53BP1 and BRCA1 spots. Class 5 structures were defined as one BRCA1 spot encapsulated by multiple 53BP1 spots. (C) Quantification of structure classes for mid and late S-phase nuclei. Mean±s.e.m. *n*=3 (4387 structures from 35 nuclei for mid S phase, 5051 structures from 39 nuclei for late S phase). (D) Class 5 structures are comprised of spot-like accumulations of 53BP1 (magenta) encapsulating the core BRCA1 spot (green). Examples of class 5 structures were selected from late S-phase nuclei. Scale bars: 2 μm, equivalent to 500 nm pre-expansion.

The cells were immunostained for 53BP1 and BRCA1 following damage by irradiation, and comparable distributions of foci were observed pre- and post-expansion (Fig. S1E). Post-expansion 3D images were deconvolved, and the organisation of 53BP1 and BRCA1 accumulations was visually investigated (examples are shown in Fig. 2A). Initial inspection suggested a heterogeneous population of 53BP1 and BRCA1 accumulations in nuclei that were classified as being in early, mid or late S phase. To quantitatively describe the spatial organisation of thousands of protein accumulations, we developed a semi-automated spot detection-based analysis, which we applied to mid and late S-phase nuclei. The number of 53BP1 and BRCA1 spots within a 2 µm radius of a core BRCA1 spot was investigated, revealing five classes of structures (Fig. 2B,C). Intriguingly, class 5 structures resembled 53BP1 and BRCA1 accumulation patterns previously described by confocal and SIM analysis of irradiated S-phase cells (Chapman et al., 2012; Kakarougkas et al., 2013), as well as some 53BP1 structures previously observed in pre- and post-replicative cells (Ochs et al., 2019) (examples shown in Fig. 2D). These data indicate that ExM can be used to visualise heterogeneous populations of protein accumulations within the nuclear architecture.

### Averaging of hundreds of nanoscale features to explore chromatin regulators

If ExM is to be routinely used for the examination of nanoscale nuclear structures, it must be capable of reporting on changes to protein distribution regulated by chromatin reorganisation. Previous observations have suggested that 53BP1 accumulations are influenced by BRCA1 (Chapman et al., 2012; Kakarougkas et al., 2013; Isono et al., 2017; Her and Bunting, 2018) and by chromatin changes mediated by SMARCAD1 and ubiquitin-specific protease 48 (USP48) (Densham et al., 2016; Uckelmann et al., 2018). SMARCAD1 has been reported to promote localisation of 53BP1 to the periphery of irradiation-induced foci, whereas USP48 restricts 53BP1 positioning in BRCA1-proficient cells.

We treated U2OS cells with siRNA to knockdown SMARCAD1 or USP48 (Fig. S2A,B) and then subjected them to ExM, staining for BRCA1 and 53BP1 (Fig. S2C,D). By eye, 53BP1 appeared to occupy smaller volumes in SMARCAD1-depleted cells and larger volumes in USP48-depleted cells (Fig. S2C,D). BRCA1 and 53BP1 structures were assigned to one of the five defined classes (Fig. S3A). In SMARCAD1-depleted cells, there were no significant changes in distributions of structures in mid S-phase cells, whereas in late S-phase cells, fewer class 5 structures, in which 53BP1 forms as a series of discontinuous spots surrounding a central BRCA1 accumulation, were observed compared to controls (Fig. S3B). In contrast, in USP48-depleted cells, we observed an increase in the number of class 5 structures in both mid and late S-phase cells (Fig. S3C).

**Fig. 3.**
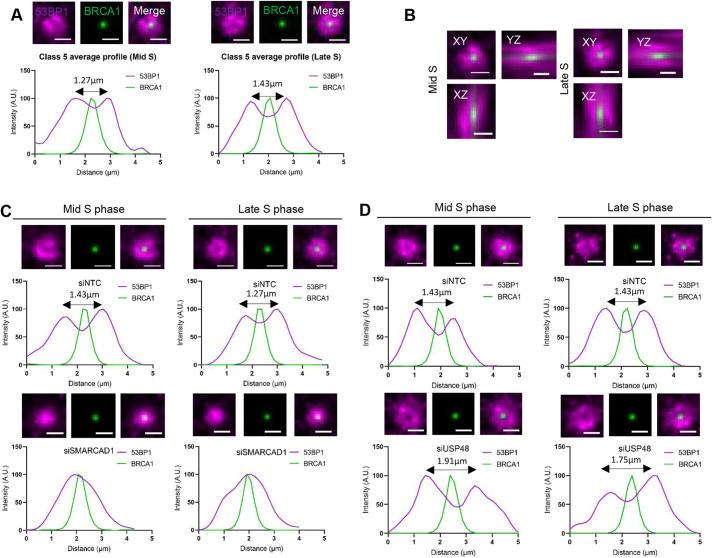
**Positioning of BRCA1 and 53BP1 following depletion of chromatin regulators SMARCAD1 and USP48.** (A) U2OS cells were treated as described in Fig. 2. Examples of class 5 structures were selected, and an average profile of the structures was generated. Images show average class 5 structures from a mid S-phase nucleus (left) and a late S-phase nucleus (right). Scale bars: 2 μm (equivalent to ∼500 nm pre-expansion). Graphs show average profiles of 53BP1 and BRCA1 in the class 5 structures (A.U., arbitrary units). *n*=3 (130 structures from 12 nuclei for mid S phase, 125 structures from 13 nuclei for late S phase). (B) Orthogonal views of average class 5 structures, as described in A, are shown. Scale bars: 2 μm. (C) U2OS cells were treated with non-target control siRNA (siNTC) or siRNA targeting SMARCAD1 (siSMARCAD1), as indicated, for 72 h. Cells were treated with EdU (not shown) and damaged with irradiation (2 Gy) before being allowed to recover for 1 h prior to fixation. Cells were immunostained for BRCA1 (green) and 53BP1 (magenta), then prepared using the ExM method. Average class 5 structures from mid S-phase nuclei and late S-phase nuclei, as indicated, are shown in the images, and average intensity profiles generated from the structures are shown in the graphs below. *n*=3 (siNTC mid S phase, 143 structures from 20 nuclei; siNTC late S phase, 184 structures from 28 nuclei; siSMARCAD1 mid S phase, 164 structures from 25 nuclei; and siSMARCAD1 late S phase, 221 structures from 31 nuclei). Scale bars: 2 μm (equivalent to ∼500 nm pre-expansion). (D) U2OS cells were treated as in C but siRNA targeting USP48 (siUSP48) was used. Average class 5 structures are shown in the images (53BP1, magenta; BRCA1, green), and average intensity profiles generated from those structures are shown in the graphs below. *n*=3 (siNTC mid S phase, 72 structures from 23 nuclei; siNTC late S phase, 77 structures from 24 nuclei; siUSP48 mid S phase, 93 structures from 22 nuclei; and siUSP48 late S phase, 135 structures from 26 nuclei). Scale bars: 2 μm (equivalent to ∼500 nm pre-expansion).

We investigated the average distribution of 53BP1 accumulations relative to the central BRCA1 spot in control cells by selecting over 100 examples of class 5 structures where proteins were oriented parallel to the focal plane, and an average structure profile was generated. In the averaged class 5 profiles, the core BRCA1 spot spanned ∼800 nm (equivalent to ∼200 nm pre-expansion). We observed that 53BP1 had a continuous localisation encapsulating BRCA1, spanning a 2.5–3 µm diameter (equivalent to 625–750 nm pre-expansion). The peak-to-peak distance of the 53BP1 distribution was measured as 1.27 µm and 1.43 µm (equivalent to 0.32 µm and 0.36 µm pre-expansion) in mid and late S-phase structures, respectively (Fig. 3A). In the orthogonal views of the averaged class 5 structure profiles, BRCA1 and 53BP1 were seen to occupy distinct regions with no visible overlap between them (Fig. 3B). From the averaged class 5 structure profiles, we estimate the separation between 53BP1 and BRCA1 to be equivalent to a pre-expansion distance of 60–80 nm (Table S1).

To examine more closely the influence of the chromatin regulators of 53BP1 using ExM, examples of tens of class 5 structures where both BRCA1 and 53BP1 were oriented approximately parallel to the focal plane were selected, and averaged structure profiles were generated from cells treated with siRNA targeting SMARCAD1 or USP48 (Fig. 3C,D). In the SMARCAD1-depleted cells, the void between the proteins was lost, and 53BP1 occupied a smaller volume, consistent with the observation that 53BP1 has a peak intensity coinciding with that of BRCA1 in the absence of SMARCAD1 (Densham et al., 2016). This observation was recapitulated in the *Z* axis (data not shown). In contrast, following USP48 siRNA treatment, a clear void was visible between the two proteins, and the 53BP1 peak-to-peak distances of 1.92 µm and 1.75 µm in mid and late S-phase average structures (equivalent to 0.48 µm and 0.44 µm pre-expansion), respectively, were increased compared to the control values of 1.43 µm (at both mid and late S phase; equivalent to 0.36 µm pre-expansion). Additionally, 53BP1 accumulations in the average structures were positioned further away from the core BRCA1, spanning ∼5 µm as compared to ∼4 µm in controls (equivalent to pre-expansion distances of 1.25 µm and 1 µm, respectively). This observation was recapitulated in the *Z* axis (data not shown). These measurements are similar to those in previously published work using confocal microscopy, where the peak-to-peak distance of 53BP1 was measured to be 0.3 μm in control cells and 0.5 μm following USP48 depletion (Uckelmann et al., 2018).

As these measurements were made on selected examples of class 5 structures (where BRCA1 and 53BP1 were oriented approximately parallel to the focal plane), we next investigated all class 5 structures of all orientations in 3D. Each class 5 structure was defined by the distance between the core BRCA1 spot and the surrounding 53BP1 accumulations (Fig. S3D,E). Following SMARCAD1 depletion, we observed that more than 70% of class 5 structures from both mid and late S-phase nuclei had a reduced distance (defined as a separation of <0.5 μm) between 53BP1 spots and the core BRCA1 spots, compared to control nuclei, where more than 85% of class 5 structures exhibited a separation distance of 1.8–2 μm between 53BP1 spots and the core BRCA1 spot. Correspondingly, in USP48-depleted cells, we observed that more than 80% of class 5 structures had an increased distance (defined as a separation of ∼2–2.5 μm) between 53BP1 spots and the core BRCA1 spot, whilst in the control nuclei, more than 70% of class 5 structures had a separation distance of ∼1.8–2 μm between 53BP1 spots and the core BRCA1 spot. To summarise, measurements undertaken on class 5 53BP1 and BRCA1 accumulations following ExM are comparable to measurements made previously using other imaging modalities, suggesting that there was minimal structural distortion in our ExM samples (Table S1).

### Four-colour ExM of nanoscale nuclear structures

**Fig. 4.**
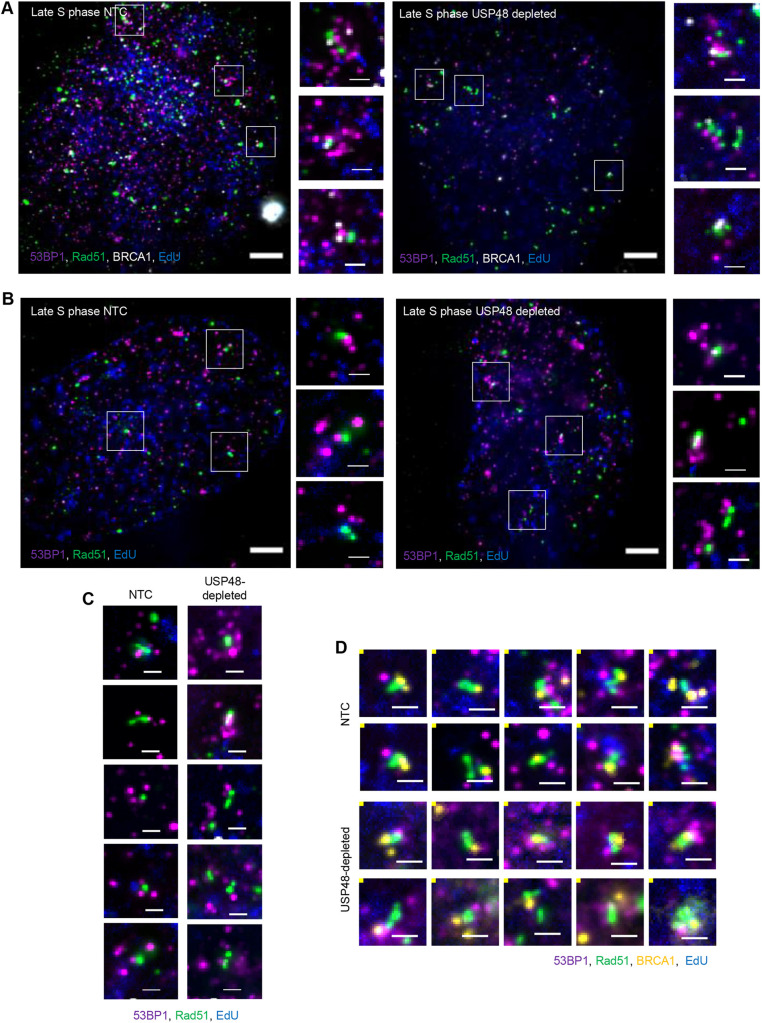
**Visualising RAD51 accumulations.** (A) U2OS cells were treated with non-target control siRNA (NTC) or siRNA targeting USP48 (USP48 depleted) for 72 h. Cells were treated with EdU (blue) prior to irradiation (2 Gy) and were then allowed to recover for 1 h prior to fixation. Cells were immunostained for RAD51 (green), BRCA1 (white) and 53BP1 (magenta) then prepared using the ExM method. Post-expansion images of late S-phase nuclei are shown. Boxes indicate regions shown in the magnified images on the right. Scale bars: 10 μm (main images) and 2 μm (selected regions), equivalent to ∼2.5 μm and 500 nm pre-expansion, respectively. (B) U2OS cells were treated with non-target control siRNA or siRNA targeting USP48 for 72 h. Cells were treated with EdU (blue) prior to irradiation (2 Gy) and were then allowed to recover for 1 h prior to fixation. Cells were immunostained for RAD51 (green) and 53BP1 (magenta), then prepared using the ExM method. Post-expansion images of late S-phase nuclei are shown. Boxes indicate regions shown in the magnified images on the right. Scale bars: 10 μm (main images) and 2 μm (selected regions), equivalent to ∼2.5 μm and 500 nm pre-expansion, respectively. (C) Examples of co-enriched structures with RAD51 (green) and 53BP1 (magenta) from late S-phase nuclei are shown. Scale bars: 2 μm (equivalent to ∼500 nm pre-expansion). (D) Examples of structures co-enriched with BRCA1 (yellow), RAD51 (green) and 53BP1 (magenta) from late S-phase nuclei are shown. Scale bars: 2 μm (equivalent to ∼500 nm pre-expansion). Images in A and C are representative of three experiments. Images in B and D are representative of two experiments.

An advantage of ExM over several other SRM methods is the ability to perform multi-colour imaging for the interrogation of several nanoscale features simultaneously. To test this capability, post-expansion 3D images of U2OS cell nuclei immunostained for BRCA1, RAD51 and 53BP1 were acquired (Fig. 4A). We examined cells treated with control siRNA or USP48 siRNA, as USP48 loss is associated with increased DNA resection lengths and increased number and intensity of RAD51 foci (Uckelmann et al., 2018). Indeed, post-expansion 3D images of USP48-depleted nuclei showed that RAD51 accumulations were visually larger (Fig. 4B,C) and that late S-phase cells had more structures classified as those with multiple RAD51 spots associated with multiple 53BP1 spots (class 4; Fig. S4A,B). We further subclassified these structures based on the presence of continuous RAD51 accumulations, and we found an increased percentage of such accumulations following USP48 depletion (Table S2).

We developed a classification approach to describe the spatial organisation in which RAD51 was arbitrarily defined as the centre of repair foci, and the relative spatial organisation of 53BP1 and/or BRCA1 relative to this centre was described. Using this method, we identified ten classes of structures (Fig. S4C,D). Of these classes, three (classes 7, 8 and 9) contained RAD51, 53BP1 and BRCA1 (Fig. S4E). In mid and late S-phase, structures defined as more than one RAD51 spot associated with multiple 53BP1 spots (class 4) were increased in number following USP48 depletion, as were late S-phase structures defined as multiple RAD51 spots associated with BRCA1 and encapsulated by multiple 53BP1 spots (class 9). We further investigated class 4 and 9 structures to assess the prevalence of continuous RAD51 accumulations within them and found increased numbers of class 4 and 9 structures with continuous RAD51 accumulations following USP48 loss (Table S3), consistent with previous observations (Uckelmann et al., 2018). We also noted that the location of BRCA1 within the class 9 structures varied; in the many selected examples, BRCA1 localised to one end of the RAD51 accumulation, whereas in other cases, BRCA1 was located at the centre of the RAD51 structure (Fig. 4D). Taken as a whole, our observations demonstrate that ExM allows for quantitative description of the spatial organisation of multiple proteins within nanoscale nuclear structures.

## DISCUSSION

Using the nucleic acid anchor LabelX in combination with approximately fourfold sample expansion, we demonstrate retention of nuclear organisation in an expanded polyacrylamide gel. The expansion factor of 3.7–4 in one dimension, which was comparable to the macroscale expansion of the gel, affords an effective resolution of ∼65–70 nm, and features in pre-expansion SIM images and post-expansion widefield images were retained between the two acquisitions with no detectable distortions.

Additionally, our findings using ExM closely correlate with those of previous assessments of protein accumulations at sites of DNA damage made using other SRM approaches. Firstly, our observation of a discontinuous spot-like appearance of 53BP1 is similar to the 53BP1 nanodomains visualised previously using STED microscopy (Ochs et al., 2019); secondly, our confirmation of chromatin-mediated regulation of the spatial relationship between 53BP1 and BRCA1, which contracted upon SMARCAD1 depletion and extended upon USP48 loss, is consistent with that previously described using confocal microscopy (Densham et al., 2016; Uckelmann et al., 2018); and finally, our observation of increased numbers of continuous RAD51 accumulations following depletion of USP48 is in agreement with a previous report (Uckelmann et al., 2018). Thus, under our methodology, nanoscale changes driven by changes in chromatin regulation are readily detectable using ExM.

Specimens prepared for ExM are optically cleared, minimising the effect of light scattering throughout the sample and allowing access to increased imaging volume and depth when compared with traditional techniques (Gao et al., 2017). This feature, together with the ability to acquire images with effectively nanoscale 3D resolution using a conventional diffraction-limited microscope, allowed us to rapidly capture thousands of protein accumulations in S-phase nuclei following irradiation. Spot detection-based analysis allowed description of spatial heterogeneity within these accumulations, demonstrating an ability to perform a complete overview of structure distribution without use bias.

We exploited the high throughput capability of ExM to explore nanoscale organisation of an unprecedented number of features, previously rendered inaccessible to other SRM techniques due to complex hardware and software requirements. Our approach allowed robust detection of changes in 53BP1 and BRCA1 accumulations following SMARCAD1 or USP48 loss, which were commensurate with those expected from previous observations (Densham et al., 2016; Uckelmann et al., 2018). These data indicate that ExM can be applied to faithfully detect nanoscale, chromatin-regulated changes within a specific nuclear architecture.

While our aim in this study was to assess the suitability of ExM for measuring nanoscale features of the nucleus, some surprising observations reported herein lead to further questions; for example, whether the heterogeneous subpopulations of 53BP1 and BRCA1 co-enriched structures relate to repair of distinct types of DNA lesions and/or chromatin states (Lomax et al., 2013), and whether the single BRCA1 spot often observed at one end of discontinuous RAD51 structures represents loading of RAD51 from one side of the DNA break. Further application of ExM using other DNA damage markers (such as phosphorylated H2AX) offers the ability to investigate these novel findings and to establish the inter-relations with chromatin and to other critical DNA repair proteins.

ExM methodology has evolved rapidly, extending the range of biomolecules that can be labelled (including lipids and sugars) and the types of labels used (Chozinski et al., 2016; Tillberg et al., 2016; Shi et al., 2021; Wen et al., 2020; Sun et al., 2021). Its resolution has been improved by combining ExM with other SRM techniques (Halpern et al., 2017; Gao et al., 2018; Xu et al., 2019), by increasing the expansion factor (Chang et al., 2017; Truckenbrodt et al., 2018) and by post-expansion labelling of biomolecules, which can improve the fidelity in the final image (Ku et al., 2016; Gambarotto et al., 2019; Faulkner et al., 2020). These have the potential to further improve the analysis of nanoscale nuclear features (Zwettler et al., 2020). Thus, with the current methods in hand, ExM can contribute significantly to a quantitative understanding of nuclear processes.

## METHODS AND MATERIALS

### Antibodies and reagents

A full list of siRNA sequences and antibodies can be found in Tables S4 and S5, respectively. Western blots show representative images taken from more than three independent experiments, unless otherwise stated. All chemicals were from Sigma or Thermo Fisher Scientific, unless otherwise stated.

### Cell lines

U2OS cells (our lab stock) were grown in Dulbecco's modified Eagle's medium (DMEM) supplemented with 10% fetal bovine serum (FBS) and 1% penicillin-streptomycin. Mycoplasma testing was performed through Hoechst 33342 DNA staining. Cells were not authenticated at the source.

### Cell growth and EdU visualisation

U2OS cells were plated at a density of 5×10^4^ cells/ml in a 24-well plate containing 13 mm #1.5 coverglasses and were treated with the thymidine analogue 5-ethynyl-2′-deoxyuridine (EdU; Life Technologies) at stated times at a concentration of 10 μM. Cells were then fixed with 4% paraformaldehyde (PFA) for 10 min at room temperature. Cells were permeabilised with 0.5% Triton X-100 in phosphate-buffered saline (PBS) for 15 min. After blocking with 10% FBS in PBS containing 0.01% Tween 20 (PBST) for 20 min, EdU staining was carried out using a Click-iT^®^ EdU imaging kit (Life Technologies) according to the manufacturer's instructions. Images were acquired prior to and following ExM preparation on widefield and selective plane illumination microscopes as stated.

### Radiation protocol

Immediately prior to irradiation, cells were treated with EdU at a final concentration of 10 μM. Cells were exposed to radiation using a Gammacell 1000 Elite irradiator (caesium-137 source) at a dose of 2 Gy. Cells were allowed to recover for 1 h in DMEM supplemented with 10% FBS and 1% penicillin-streptomycin.

### Correlative imaging

U2OS cells were treated as described above. Following digestion of ExM samples (see below), gels were cut into a distinctive shape for orientation of samples, and images were acquired pre-expansion on a structured illumination microscope. Specimens were then expanded by addition of water, and post-expansion images of the same nuclei were acquired on a widefield microscope.

### Transfection

siRNA transfections were carried out using the transfection reagent DharmaFECT 1 (Dharmacon) according to the manufacturer's instructions.

### Immunofluorescence staining and discrimination of S-phase nuclei

Cells were subject to immunofluorescence staining prior to standard microscopy using a widefield microscope or were prepared using ExM methodology (see below). Cells were plated at a density of cells 3×10^4^ cells/ml in a 24-well plate containing 13 mm #1.5 coverglasses and were treated as required. Cells were pre-extracted with CSK buffer (100 mM sodium chloride, 300 mM sucrose, 3 mM magnesium chloride and 10 mM PIPES, pH 6.8) for 1 min at room temperature. Cells were fixed in 4% PFA for 10 min at room temperature and permeabilised with 0.5% Triton X-100 in PBS for 15 min at room temperature. After blocking with 10% FBS, EdU staining was carried out using a Click-iT^®^ EdU imaging kit (Life Technologies). EdU incorporation can be detected by reaction with a fluorescent azide dye in a copper (I)-catalysed azide–alkyne cycloaddition (Salic and Mitchison, 2008). EdU incorporation resulted in well-defined patterns of incorporation that allowed discrimination between early, mid and late S-phase cells. Azide dyes used for EdU detection were Alexa Fluor 488 (C10337, Life Technologies), AZDye 405 (1307, Click Chemistry Tools) and Alexa Fluor 647 (C10340, Life Technologies). Cells were incubated with primary antibodies at the stated concentrations for either 1 h at room temperature or overnight at 4°C, and with the secondary Alexa Fluor-conjugated antibodies for 1 h at room temperature (summarised in Table S5).

### Sample Expansion

#### Anchor synthesis

Acryloyl-X [6-((acryloyl)amino)hexanoic acid, succinimidyl ester; AcX; A20770, Thermo Fisher Scientific] was resuspended in anhydrous DMSO with a final concentration of 10 mg/ml. This was then aliquoted and stored in a frozen desiccated environment for up to 2 months. Label-IT amine (MIR3900, Mirus Bio; 100 µg) was resuspended in reconstitution solution (100 µl) with a final concentration of 1 mg/ml. To produce LabelX, 10 µl of AcX was added to label-IT amine and reacted overnight with shaking at room temperature. This was subsequently aliquoted and stored in a frozen desiccated environment for up to 2 months.

#### Anchoring of cellular DNA and proteins

Cells were washed with 20 mM MOPS pH7.7 and incubated with the nucleic acid anchor LabelX (at a final concentration of 0.006 mg/ml) in MOPS at 37°C overnight. Following two washes with PBS, cells were incubated with the protein anchor AcX (0.1 mg/ml) in PBS for >6 h at room temperature. Specimens were washed with PBS prior to gelation.

#### Gelation, digestion and expansion

Monomer solution [PBS containing 2 M NaCl, 8.625% (w/w) sodium acrylate (97%, 744-81-3, Sigma Aldrich), 2.5% (w/w) acrylamide (79-06-1, Sigma Aldrich) and 0.15% (w/w) N,N′-methylenebisacrylamide (110-26-9, Sigma Aldrich)] was mixed, frozen in aliquots and thawed prior to use. Concentrated stocks of ammonium persulfate (APS; 7727-54-0, Sigma Aldrich) and tetramethylethylenediamine (TEMED; 110-18-9, Sigma Aldrich) at 10% (w/w) in water were diluted into the monomer solution to concentrations of 0.2% (w/w) on ice prior to gelation, with the initiator (APS) added last. The gelation solution (80 µl) was placed on a parafilm-covered slide in a humidified chamber. Coverslips were inverted onto the droplet with the cells face down. Gelation was allowed to proceed at 37°C for 2 h in a humidified chamber. Gels were removed from the slide and immersed in digestion buffer (1× TAE with 0.5% Triton X-100 and 0.8 M guanidine HCl) containing 8 units/ml Proteinase K (P8107S, New England Biolabs Inc.) was added freshly to the digestion buffer. Gels were digested either at room temperature overnight or at 37°C for 4 h. The gels were removed from the digestion buffer and placed in 50 ml of water to expand. Water was exchanged every 30 min until expansion was complete (typically three or four exchanges).

### Expanded specimen handling for imaging

For 3D-SIM imaging, unexpanded gels were mounted on high tolerance #1.5 Ibidi glass-bottomed dishes (Thistle Scientific, IB-81158). For widefield imaging, expanded gels were cut to fit in MatTek dishes with glass coverslips of 35 mm diameter (MatTeK Life Sciences, P35G-1.5-14-C). Excess water was removed, and gels were embedded in 2% low melting point (LMP) agarose to limit gel movement during image acquisition. For selective plane illumination microscopy (SPIM) imaging, gels were cut to fit the SPIM holder and placed cell-side up in the SPIM holder. Next, 2% LMP agarose was pipetted into the holder until the bottom of the holder was covered, taking care not to get agarose in the interface between the top of the ExM gel and the objective lens. Deionised water was then added to the SPIM holder containing the gel to fully immerse the gel for imaging (details below).

Post-expansion images of nuclei were acquired on a SPIM to enable good optical sectioning with minimal photodamage of the specimen (Huisken and Stainier, 2009). Specimens prepared for ExM are optically transparent due to the large amount of water absorbed by the polymer, meaning the gels are refractively matched to the water immersion medium and objectives required by the SPIM (Gao et al., 2017). These features minimised optical aberrations, and minimal processing was required to visualise nanoscale features of the nuclear architecture.

### Image acquisition

Structured illumination microscopy (SIM) was performed on a Nikon N-SIM-S system (Ti-2 stand; Hamamatsu ORCA Flash 4.0 scientific CMOS dual cameras with Cairn splitter system; Nikon Perfect Focus; Chroma ET525/50m, ET595/50m and ET 700/75m emission filters; and Nikon laser bed with 405 nm/488 nm/561 nm/640 nm laser lines). A Nikon 100×1.49 NA TIRF oil objective was used. NIS Elements v5 software (Nikon) was used to control the system and acquire pre-expansion 3D-SIM images. Expanded samples were imaged on an ASI RAMM microscope frame. Widefield imaging was performed using a Nikon 100× TIRF (NA 1.45) objective and an Evolve Delta EM-CCD camera, via a quad-band emission filter (Semrock, 432/515/595/730 nm). iSPIM was performed using twin Nikon 40× (NA 0.8) water-dipping objectives, a similar quad-band emission filter (Semrock, 432/515/595/730 nm) and a Hamamatsu ORCA Flash 4.0 scientific CMOS camera. Illumination for both setups was from a Cairn Research laser bank containing 100 mW 405 nm, 150 mW 488 nm, 50 mW 561 nm and 100 mW OBIS 640 nm continuous wave (CW) lasers. Light was directed to the sample via a quad-band dichroic mirror (Semrock, 405/488/561/635 nm). Micro-Manager (https://micro-manager.org/) was used to control the system and scan the sample.

### Image processing

Images acquired on the Nikon N-SIM-S system were reconstructed using stack reconstruction in the NIS elements software. Where stated, post-expansion image data was deconvolved using Huygens professional version 19.04 (Scientific Volume Imaging, The Netherlands; http://svi.nl). A theoretical point spread function (PSF) was generated based on the microscope parameters, and images were deconvolved using a classical maximum likelihood estimation (CMLE), a non-linear iterative restoration method that optimises the likelihood the objects in the estimated image are correctly localised based on the image and the PSF. This restoration method relies on the generation of an estimate of an object (synthetic image), which is compared to the measured image. The result of this is used to improve the original until the ‘difference’ between the synthetic and measured image reach a minimum. Parameters for deconvolution were tested on example data sets for each experiment to determine optimal values, and these deconvolution templates were used for subsequent image processing and experimental repeats.

### Segmentation of nuclei

Quantification of nuclear areas pre- and post-expansion was performed using a script written in MATLAB (MathWorks). Briefly, pre- and post-expansion images were processed by wperforming a rolling ball background subtraction, and .tif files were saved into corresponding directories. Images were segmented using a manually determined threshold based on histograms generated from the images. Nuclei were segmented, resulting in a mask of pixels where fluorescence was above the manually determined threshold. Boundaries were traced onto the binary image, and then these boundaries were superimposed onto the original image to determine the efficacy of segmentation. The centroid of each nucleus was determined and labelled. The areas of the labelled objects were calculated in pixels and square micrometres (μm^2^). Optimal parameters were determined and then used to determine nuclear areas pre- and post-expansion. A maximum and minimum area was defined based on the assumption that nuclei roughly conform to a circle, to remove features too small to be nuclei and features that corresponded to more than one nucleus.

For determination of nuclear volumes pre- and post-expansion, images were segmented using auto-threshold in ImageJ (NIH, Bethesda, MD) to generate a mask. Otsu threshold efficiently segmented nuclei from background pixels. The generated mask was eroded and dilated, and any gaps were filled in. The volumes of the final masks were measured for each image. For each image, an object map was generated and compared to the original images to determine the efficacy of the segmentation.

### Registration of pre-expansion SIM images and post-expansion images

Registration of pre-expansion SIM and post-expansion widefield images was performed as follows. Twenty-one control points were manually selected at the same positions in 3D for both the expanded widefield data and the SIM data using a customised MATLAB (R2020b) graphical user interface. Next, 80% of control points were used to fit a similarity registration transform (translation, rotation and scaling) using the ABSOR MATLAB function, which is based on Horn's quaternion-based algorithm (Horn et al., 1988). The remaining 20% of points were kept back for validation purposes. The MSE between pairwise control points was found to be 172 nm and 282 nm for the training and validation points, respectively. The widefield and SIM data were resized to have equal isotropic voxel size (29.8 nm) using bi-cubic interpolation before applying the similarity transformation to the widefield data to produce a two-channel registered volume.

### Spot-detection-based analysis of nuclear structures

Spot detection-based analysis of structures was performed using customised Groovy script written for the open-source application Fiji (Schindelin et al., 2012) available at https://github.com/JeremyPike/expansion-analysis. First, spots were detected in each channel of interest by finding local maxima in a Laplacian of Gaussian-filtered volume. Local maxima were filtered based on the prominence of each maximum relative to its local neighbourhood (spot quality). Spot detection was implemented using the TrackMate (Tinevez et al., 2017) plugin. Spots in the assigned central site channel (e.g. BRCA1) were then clustered by finding the connected components of the graph formed by linking all spots within a fixed radius. Cropped 3D volumes centred on the centre of mass for each cluster were presented to the user to manually classify the type of repair foci structures. In this work, we analysed foci structures containing two or three repair proteins simultaneously.

The general workflow for spot detection-based analysis of nanoscale nuclear structures involved the following steps: (1) all parameters were defined (as summarised in Tables S6 and S7); (2) spot detection was performed as described above, according to the parameters set, and detections were displayed in 3D in the whole nucleus; (3) we determined whether an image was suitable for subsequent classification of structures – images were omitted if sample movement or photobleaching was evident in the images; (4) each feature (defined by the presence of a spot in the site channel) was displayed in the crop box, which had a size of 5 μm^3^, and classified.

All experiments and subsequent spot detection-based analysis parameters are shown in Table S7. In all experiments, cells were treated with EdU prior to irradiation (2 Gy) and allowed to recover for 1 h.

### Generation of average foci structures and colocalisation analysis

To generate average structures of class 5 structures, examples of structures were selected where BRCA1 and 53BP1 were orientated parallel to the focal plane. These example structures were selected by visualising each class 5 structure in 3D using the spot detection-based algorithm. The example structures were organised into directories, and then an average structure was generated using a customised MATLAB script available at https://github.com/JeremyPike/expansion-analysis. The script generated the average structures as 3D image stacks, which were used to generate the maximum projections and orthogonal views. Additionally, the script generated a radial profile for each channel in the average structure. The radial profile was produced by binning pixels in the central slice into bands of varying distance (fixed width) from the site centre. The average intensity of all pixels in the band determined the radial profile at a specific distance.

### Defining the position of 53BP1 accumulations relative to core BRCA1 spot

To describe the placement of 53BP1 accumulations relative to the core BRCA1 spot following USP48 or SMARCAD1 depletion, all class 5 structures for mid and late S-phase classified nuclei were subclassified according to the distance of 53BP1 accumulations from the central BRCA1. These definitions are supplied in Table S8.

## Supplementary Material

Supplementary information

Reviewer comments
