# Supplementary material for: Imaging nanoscale nuclear structures with expansion microscopy
Source: J Cell Sci. 2022 Jul 19;135(14):jcs259009. doi: 10.1242/jcs.259009 (PMC9450888; doi:10.1242/jcs.259009)
Supplement: Supplementary information [file joces-135-259009-s1.pdf]

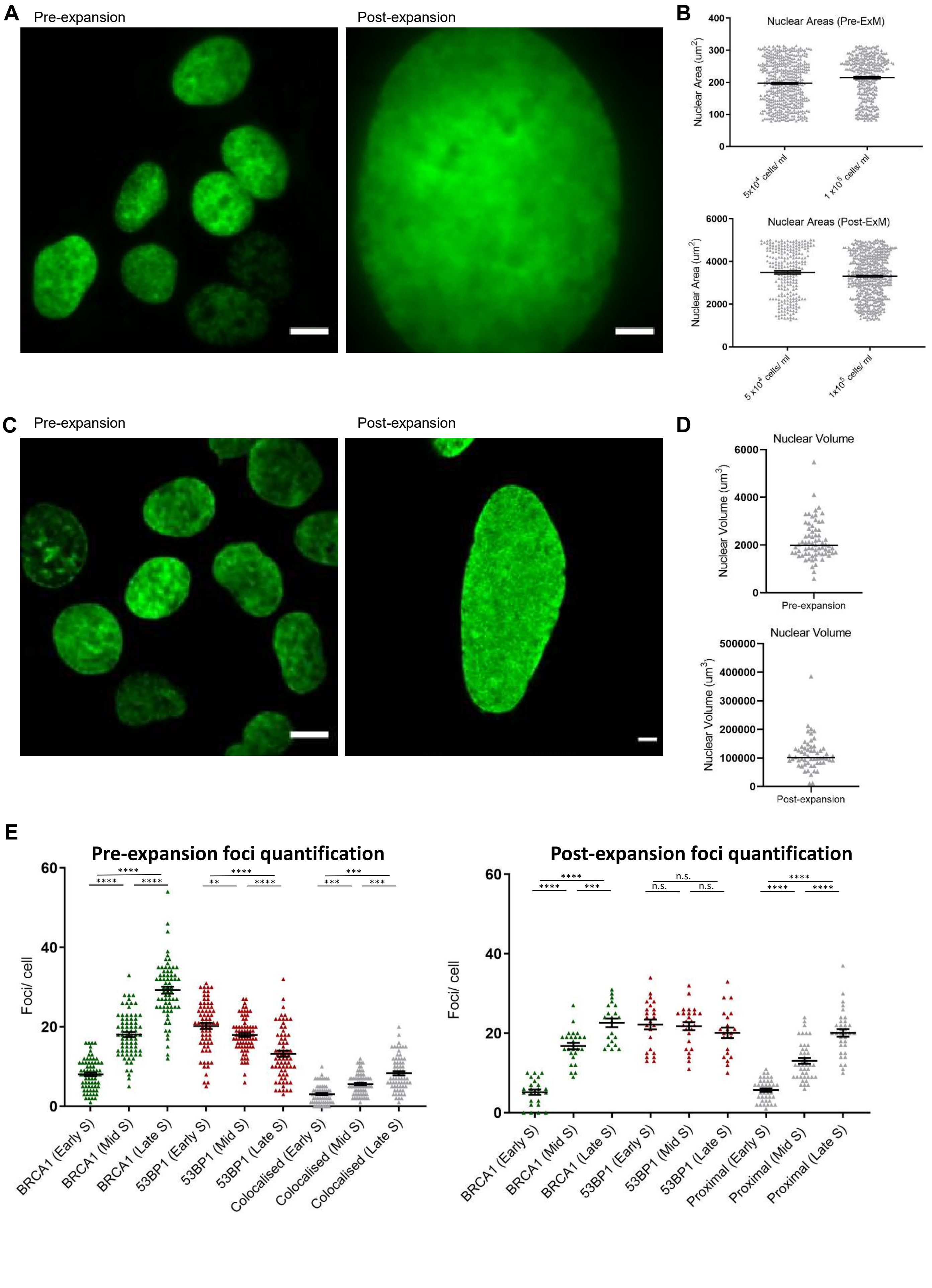

Journal of Cell Science • Supplementary information

Fig. S1. Isotropic expansion of the nucleus.

A. U2OS cells were treated with EdU overnight prior to fixation. Click it reaction was performed for detection of EdU and images were acquired either pre-expansion or post-expansion. 2D images of nuclei were acquired pre- and post-ExM. EdU is shown in green. Scale bars 10 µm.

B. Nuclear areas were quantified by segmenting nuclei from background pixels. N=3, mean ± s.e.m.

C. U2OS cells were treated as in Fig. S1A. 3D images of nuclei were acquired pre-ExM on a widefield microscope and deconvolved. Post-ExM images of nuclei were acquired on a SPIM. Images are shown as maximum intensity projections. EdU is shown in green. Scale bars 10 µm and 40 µm, respectively.

D. Nuclear volumes were quantified pre- and post-ExM. N=3, mean ± s.e.m.

E. U2OS cells were treated with EdU prior to damage with irradiation (2 Gy) and allowed 1 hour to recover prior to fixation. Cells were immunostained for BRCA1 and 53BP1, then images were acquired pre-ExM or samples were prepared using ExM method. EdU incorporation was used to classify nuclei as early, mid or late S phase and foci quantification was performed. In post-expansion Z stacks, foci numbers were counted in 3D. In pre-expansion images, colocalisation was determined by visualising overlapping pixels in foci containing both proteins. Colocalisation was not observed in expanded nuclei. Proximal structures are defined as those where 53BP1 and BRCA1 are present within 1 µm of each other in any dimension. N=3, mean ± s.e.m. ****P<0.001; ***P < 0.005; **P <0.01;*P < 0.05 NS, not significant by two-tailed Student's t test.

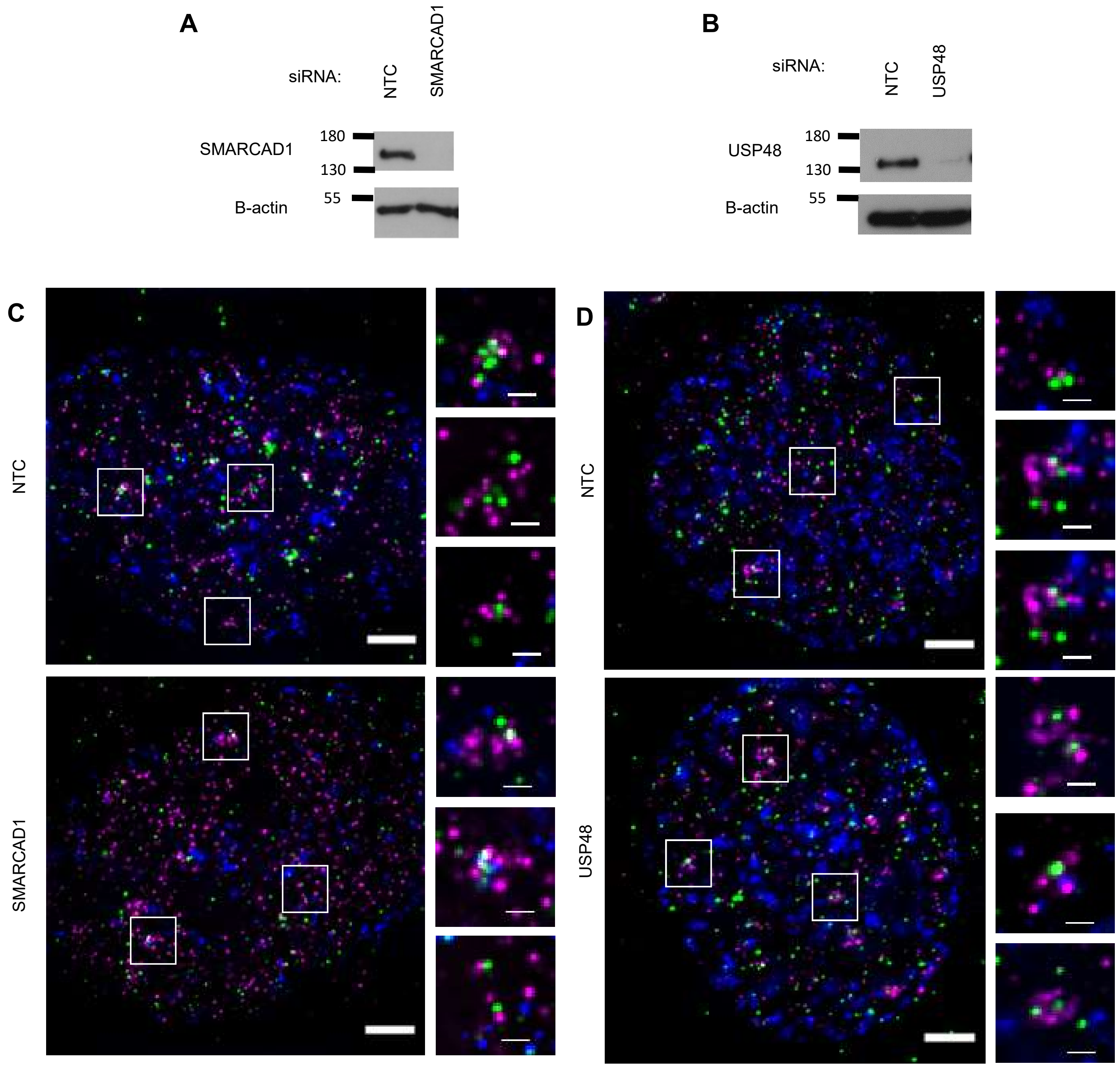

Fig. S2. 53BP1:BRCA1 imaging following depletion of chromatin regulators, SMARCAD1 and USP48.

A. U2OS cells were treated with siNTC or siSMARCAD1 for 72 hours prior to lysis. SMARCAD1 depletion was confirmed using antibodies shown.

B. U2OS cells were treated with siNTC or siUSP48 for 72 hours prior to lysis. USP48 depletion was confirmed using antibodies shown.

C. U2OS cells were treated with siNTC or siSMARCAD1 for 72 hours. Cells were treated with EdU (blue) for 1 hour prior to irradiation (2 Gy) and allowed 1 hour to recover prior to fixation. Cells were immunostained for BRCA1 (green) and 53BP1 (magenta), then prepared using ExM method. Post-expansion images late S-phase classified nuclei were acquired. Scale bars 10 µm (large images) and 2 µm (selected features), equivalent to ~2.5 µm and 500 nm pre-ExM, respectively.

D. U2OS cells were treated with siNTC or siUSP48 for 72 hours. Cells were treated with EdU (blue) for 1 hour prior to irradiation (2 Gy) and allowed 1 hour to recover prior to fixation. Cells were immunostained for BRCA1 (green) and 53BP1 (magenta), then prepared using ExM method. Post-expansion images late S-phase classified nuclei were acquired. Scale bars 10 µm (large images) and 2 µm (selected features) equivalent to ~2.5 µm and 500 nm pre-ExM, respectively.

Journal of Cell Science • Supplementary information

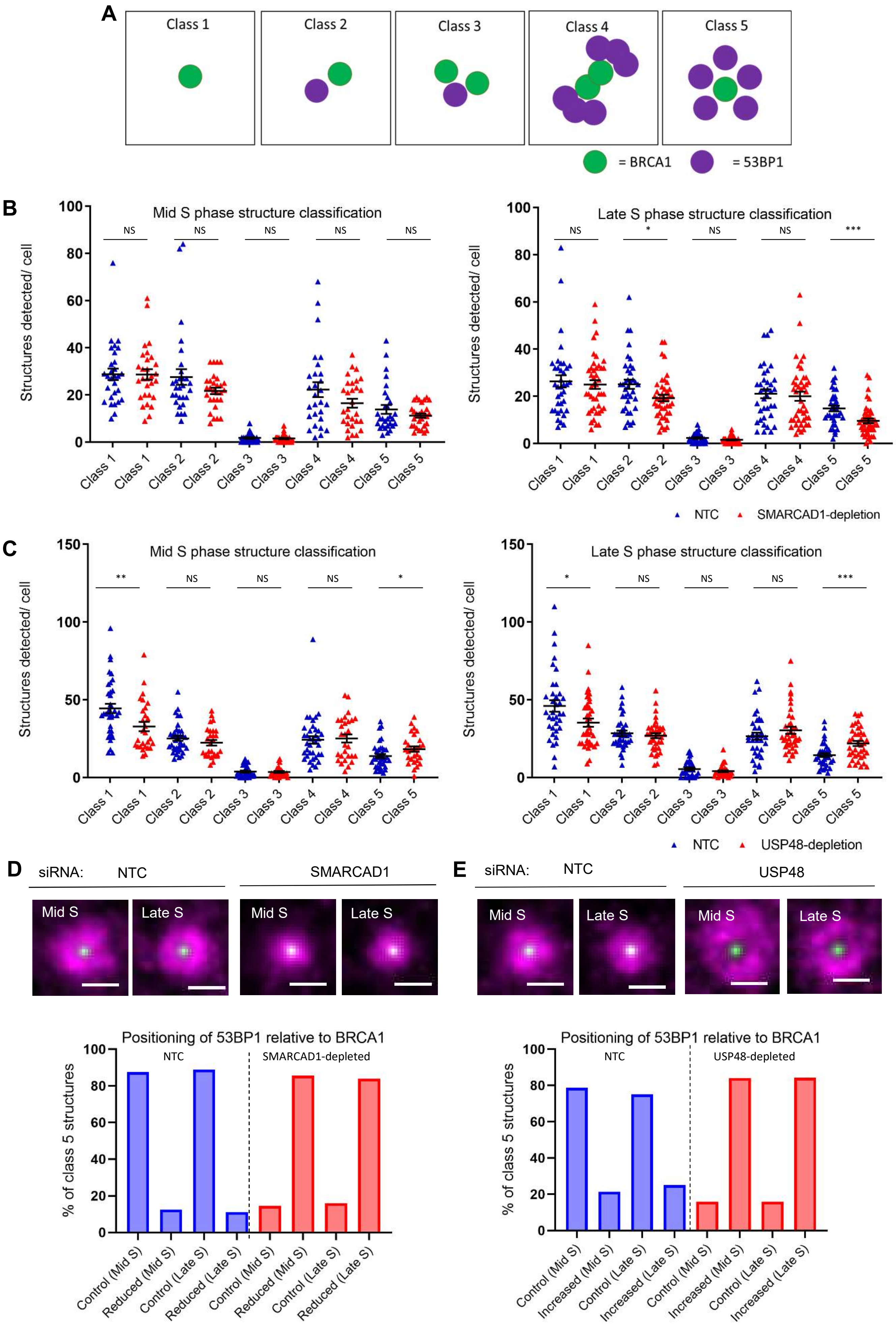
A
Class 1
Class 2
Class 3
Class 4
Class 5
= BRCA1
= 53BP1
B
Mid S phase structure classification
Late S phase structure classification
Structures detected/ cell
NS
*
***
Class 1
Class 2
Class 3
Class 4
Class 5
NTC
SMARCAD1-depletion
C
Mid S phase structure classification
Late S phase structure classification
Structures detected/ cell
**
NS
*
***
NTC
USP48-depletion
D
siRNA:
NTC
SMARCAD1
Mid S
Late S
E
siRNA:
NTC
USP48
Mid S
Late S
Positioning of 53BP1 relative to BRCA1
NTC
SMARCAD1-depleted
USP48-depleted
% of class 5 structures
Control (Mid S)
Reduced (Mid S)
Control (Late S)
Reduced (Late S)
Increased (Mid S)
Increased (Late S)

Fig. S3. **The spatial organisation of thousands of nanoscale BRCA1:53BP1 features after depletion of chromatin regulators SMARCAD1 and USP48.**

A. Schematic representation of structure classes 1-5 co-enriched with BRCA1 (green) and 53BP1 (purple).

B. Quantification of structure classes in mid and late S-phase cells following SMARCAD1 depletion (red) compared to controls (blue), n=3, mean ± s.e.m. (NTC mid S = 29 nuclei, 2712 structures, NTC late S = 37 nuclei, 3320 structures, SMARCAD1 mid S = 29 nuclei, 2312 structures, SMARCAD1 late S = 43 nuclei, 3242 structures). ***P < 0.005; *P < 0.05 NS, not significant by two-tailed Student's t test.

C. Quantification of structure classes in mid and late S-phase cells following USP48 depletion (red) compared to controls (blue), n=3, mean ± s.e.m. (NTC mid S = 37 nuclei, 4133 structures, NTC late S = 36 nuclei, 4357 structures, USP48 mid S = 28 nuclei, 2867 structures, USP48 late S = 38 nuclei, 4515 structures). ***P < 0.005; **P <0.01;*P < 0.05 NS, not significant by two-tailed Student's t test.

D. All class 5 structures in siSMARCAD1 and siNTC treated cells were classified as having a reduced separation between BRCA1 and 53BP1 (defined as <0.5 µm) or a separation distance of ~1.8-2 µm (referred to as a control separation distance as determined in Figure 3A), respectively. Average class 5 structures were generated (shown as maximum projections, scale bar 2 µm), and the percentage of class 5 structures classified as having normal or reduced separation are shown. N=3 (NTC mid S = 360 structures from 29 nuclei, NTC late S = 469 structures from 37 nuclei, SMARCAD1-depletion mid S = 298 structures from 29 nuclei, SMARCAD1-depletion late S = 311 structures from 43 nuclei).

E. All class 5 structures in siUSP48 and siNTC treated cells were classified as having an increased separation between BRCA1 and 53BP1 of ~2-2.5 µm or a separation distance of ~1.8-2 µm (referred to as a control separation distance as determined in Figure 3A), respectively. Average class 5 structures were generated (shown as maximum projections, scale bar 2 µm) and the percentage of class 5 structures classified as having normal or increased separation are shown. N=3 (NTC mid S = 414 structures from 37 nuclei, NTC late S = 384 structures from 36 nuclei, USP48-depletion mid S = 404 structures from 28 nuclei, USP48-depletion late S = 609 structures from 38 nuclei).

Journal of Cell Science • Supplementary information

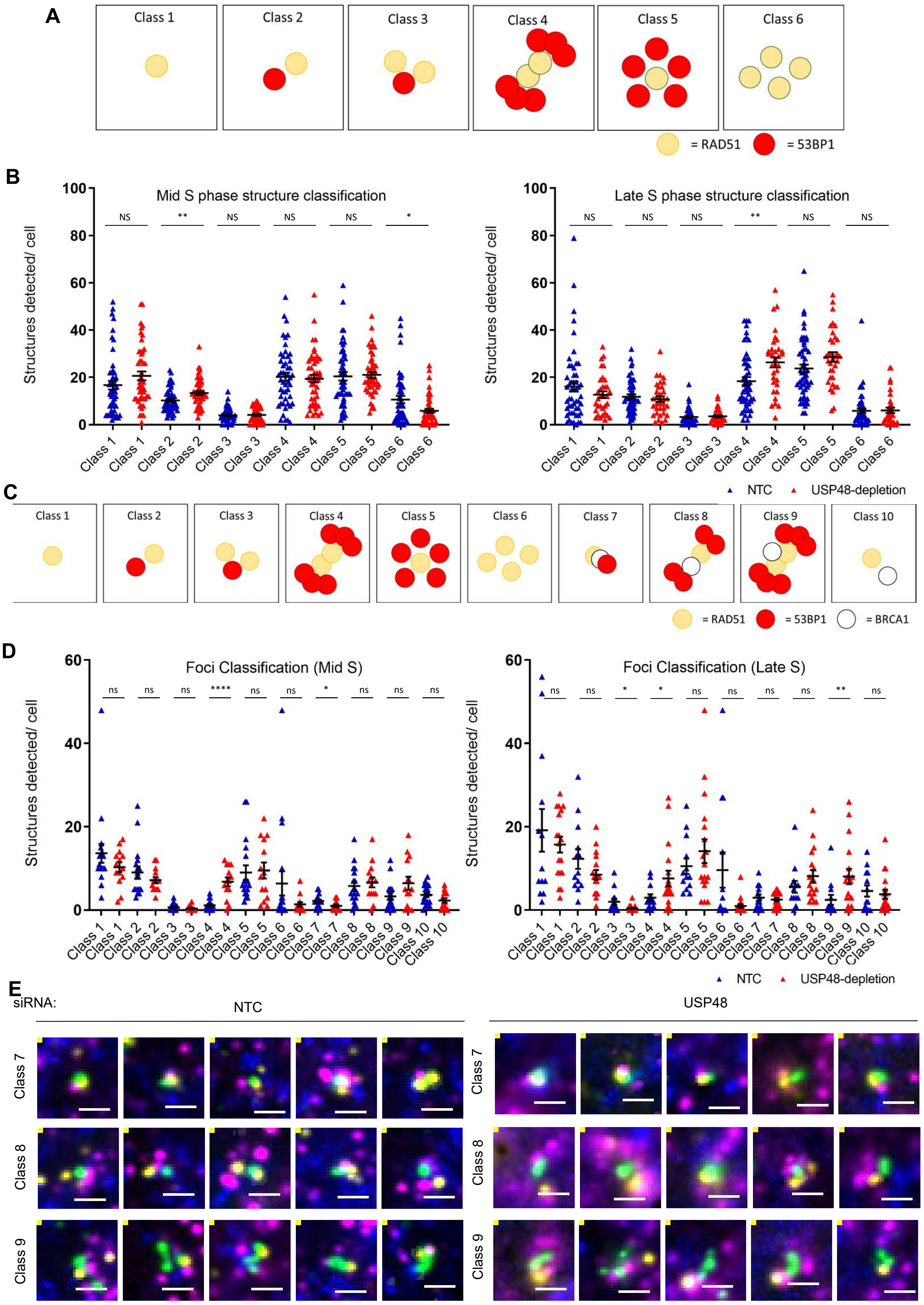

Journal of Cell Science • Supplementary information

Fig. S4. The spatial organisation of thousands of nanoscale RAD51, 53BP1 and BRCA1 features following USP48 depletion. U2OS cells were treated with either control or USP48 siRNA for 72 hours. Cells were treated with EdU (blue) prior to damage induction with irradiation (2 Gy) and allowed 1 hour to recover prior to fixation. Cells were immunostained for RAD51 (green), BRCA1 (yellow) and 53BP1 (magenta) and then prepared using ExM.

A. Schematic representation of structure classes 1-6 enriched with RAD51 (yellow) and 53BP1 (red).

B. Quantification of structure classes in mid and late S-phase cells in siUSP48 and siNTC treated cells, n=3, mean ± s.e.m. (NTC mid S = 51 nuclei, 4156 structures, USP48-depleted mid S = 54 nuclei, 4118 structures, NTC late S = 48 nuclei, 4054 structures, USP48-depleted late S = 34 nuclei, 2992 structures). **P <0.01;*P < 0.05 NS, not significant by two-tailed Student's t test.

C. Schematic representation of structure classes 1-10 with RAD51 (yellow), BRCA1 (white) and 53BP1 (red).

D. Quantification of structure classes in mid and late S-phase cells following USP48 depletion compared to controls, n=2, mean ± s.e.m. (NTC mid S = 19 nuclei, 983 structures, USP48-depleted mid S = 14 nuclei, 725 structures, NTC late S = 13 nuclei, 940 structures, USP48-depleted 1260 structures) **** P<0.001; **P <0.01;*P < 0.05 NS, not significant by two-sided Student's t test.

E. Examples of class 7 (defined as BRCA1, RAD51 and 53BP1 spots which are closely associated), class 8 (defined as 1 RAD51 spot and 1 BRCA1 spot encapsulated by multiple 53BP1 spots) and class 9 (defined as multiple RAD51 spots with BRCA1 spot(s) associated and encapsulated by multiple 53BP1 spots) structures selected from late S-phase classified nuclei are shown for USP48 depleted cells and controls. Scale bars 2µm (equivalent to ~500nm pre-ExM).

Journal of Cell Science • Supplementary information

Table S1. Summary of 53BP1:BRCA1 foci measurements following SMARCAD1 and USP48 depletion.

**Condition** [i]	**53BP1 peak-to-peak distance (μm)**	**BRCA1 spot diameter (μm)**	**Approximate separation distance between 53BP1 and BRCA1 (μm)**[ii]
NTC (mid S)	1.43 (0.35 pre-ExM)[iii]	0.8 (0.2 pre-ExM)	0.32 (0.079 pre-ExM)
NTC (late S)	1.27 (0.32 pre-ExM)[3]	0.8 (0.2 pre-ExM)	0.24 (0.058 pre-ExM)
SMARCAD1 depletion (mid S)	NA[iv]	0.8 (0.2 pre-ExM)	NA
SMARCAD1 depletion (late S)	NA[iv]	0.8 (0.2 pre-ExM)	NA
NTC (mid S)	1.43 (0.35 pre-ExM) [3]	0.8 (0.2 pre-ExM)	0.32 (0.079 pre-ExM)
NTC (late S)	1.43 (0.35 pre-ExM) [3]	0.8 (0.2 pre-ExM)	0.32 (0.079 pre-ExM)
SMARCAD1 depletion (mid S)	1.91 (0.48 pre-ExM)[v]	0.8 (0.2 pre-ExM)	0.56 (0.139 pre-ExM)
SMARCAD1 depletion (late S)	1.75 (0.44 pre-ExM)[5]	0.8 (0.2 pre-ExM)	0.48 (0.119 pre-ExM)

[i] Pre-ExM measurements determined by dividing by expansion factor determined in Fig. 1 and Fig. S1.
[ii] Calculated by subtracting BRCA1 foci diameter from 53BP1 peak-to-peak distance and dividing the result by 2 (denoting separation on either side).
[iii] Compared to 0.3 μm determined using confocal microscopy (Uckelmann *et al*, 2018) & 0.75 μm determined using confocal microscopy (Kakarougas *et al*, 2013) (N.B. measured with RPA not BRCA1).
[iv] Compared to no separation determined using confocal microscopy (Densham *et al*¸ 2016).
[v] Compared to 0.5 μm determined using confocal microscopy (Uckelmann *et al*, 2018)

Table S2. Sub-classification of RAD51 accumulations following USP48 depletion.

Condition[vi]	Subclass 1[vii] Percentage	Subclass 2[viii] Percentage	Subclass 3[ix] Percentage
NTC (mid S)	41.28	21.18	37.54
NTC (late S)	49.88	21.95	28.18
USP48-depleted (mid S)	53.67	19.11	27.22
USP48 depleted (late S)	57.80	17.99	24.21

[vi] Percentages derived from NTC mid S = 642 structures from 51 nuclei, NTC late S = 401 structures from 54 nuclei, USP48-depleted mid S = 518 structures from 48 nuclei, USP48-depleted late S = 756 structures from 34 nuclei
[vii] defined as continuous RAD51 structures encapsulated by multiple 53BP1 spots within a 2 μm radius
[viii] defined as discontinuous RAD51 structures encapsulated by multiple 53BP1 spots within a 2 μm radius
[ix] defined as multiple RAD51 spots associated with multiple 53BP1 spots within a 2 μm radius

Table S3. Number of class 4 and class 9 structures with a continuous RAD51 structure following USP48 depletion.

Condition	Class 4 structures with continuous RAD51 (>1.5 µm[x])	Class 9 structures with continuous RAD51 (>1.5 µm)
**NTC (mid S)**	22	37
**NTC (late S)**	22	27
**USP48-depleted (mid S)**	64	60
**USP48-depleted (late S)**	100	93

[x] equivalent to >0.375 µm pre-expansion

Table S4. siRNA sequences.

siRNA Name	5'-3' Sequence
NTC (Renilla Luciferase)	Sense: CUUACGCUGAGUACUUCGA[dT][dT]<br>Antisense: [Phos]UCGAAGUACUCAGCGUAA G[dT][dT]
SMARCAD1 #1	Sense: GAC GAU UGA AGA AUC CAU GCU [dTdT]<br>Antisense: [Phos] AGC AUG GAU UCU UCA AUC GUC [dTdT]
SMARCAD1 #2	Sense: AUG UAG UUA UAA GGC UUA UGA [dTdT]<br>Antisense: [Phos] UCA UAA GCC UUA UAA CUA CAU [dTdT]
USP48 Exon 5	Sense: GCGUAAGCAAAGUGUGGAUAA[dT][dT]<br>Antisense: [Phos]UUAUCCACACUUUGCUUACGC[dT][dT]
USP48 Exon 11	Sense: GAAUCCAGAUGUGCGCAAUAU[dT][dT]<br>Antisense: [Phos]AUAUUGCGCACAUCUGGAUUC[dT][dT]

Journal of Cell Science • Supplementary information

Table S5. Antibodies including species raised in, concentration, conditions and protocols.

Antibody	Animal	Procedure	Concentration	Time	Supplier & Cat. number
53BP1	Goat	IF	1:5000	1 hour	R&D systems Af1877
β-actin	Rabbit	WB	1:5000	Overnight	Abcam Ab8227
BRCA1 (D9)	Mouse	IF	1:500	Overnight	Santa Cruz Sc6954
Lamin B1	Rabbit	WB	1:3000	Overnight	Abcam Ab16048
RAD51	Rabbit	IF	1:1000	Overnight	Santa Cruz SC8349
SMARCAD1	Rabbit	WB	1:1000	Overnight	Bethyl a301-593a-m
USP48	Rabbit	WB	1:1000	Overnight	Abcam Ab72226
Donkey α Mouse AlexaFluor 488	Donkey	IF	1:5000	1 hour	Life technologies A21202
Donkey α Goat CF 633	Donkey	IF	1:5000	1 hour	Biotium 20127
Donkey α Mouse AlexaFluor 568	Donkey	IF	1:5000	1 hour	Life technologies A10037
Donkey α Rabbit AlexaFluor 488	Donkey	IF	1:5000	1 hour	Life technologies A32790
Donkey α goat AlexaFluor 568	Donkey	IF	1:5000	1 hour	Life technologies A-11057
Rabbit α Mouse HRP	Rabbit	WB	1:10,000	1 hour	Dako P0161
Swine α Rabbit HRP	Swine	WB	1: 10,000	1 hour	Dako P0217

Table S6. User defined parameters for spot detection-based analysis.

User defined parameter	Explanation
**Input directory**	File path containing the images, file extension is defined (e.g. tif). EdU incorporation was used to classify images according to the cell cycle phase and images were placed into directories accordingly. We used mid and late S-phase classified nuclei in all experiments.
**Voxel size**	Given in microns.
**Spot radius**	Selected for each channel and given in microns.
**Quality value**	This is measure of maxima prominence set within Trackmate. The quality values of the detected spots are displayed as a histogram in Trackmate. We defined an average quality threshold for each channel using training data sets of nuclei immunostained for repair proteins (e.g. BRCA1, 53BP1) to ensure relevant spots were detected.
**Rolling ball subtraction**	Defined for each channel based on the maximum size of features in the channels to remove any background fluorescence which had not been removed by deconvolution of images.
**Site channel**	Also referred to as the central channel and was defined as the channel containing the features to be treated as the core of structures of interest.
**Satellite channels**	Any other channels where spot detection is carried out, up to two used in this work.
**Crop box size**	Defined in microns, each feature of interest was displayed in this crop box in 3D for classification.
**Number of classes**	Number of spatial relationships observed between proteins. We defined this based on visual inspection of features in training data sets for each experiment.

Journal of Cell Science • Supplementary information

Table S7. Summary of analysis parameters used to analyse BRCA1, 53BP1 and RAD51 accumulations in mid and late S-phase classified cells.

Experiment[xi]	siRNA	Primary antibodies	Spot detection-based analysis script	Central spot	Satellite spot(s)	Spot radius (µm)	Quality value	Rolling ball subtraction values (pixels)	Number of classes identified
Investigating BRCA1 and 53BP1 accumulations	N/A	BRCA1, 53BP1	Two-colour analysis	BRCA1	53BP1	0.75	7.5 (53BP1)<br>6 (BRCA1)	40 (53BP1)<br>14 (BRCA1)	5
Investigating BRCA1 and 53BP1 accumulations following SMARCAD1 and USP48 loss	siSMARCAD1<br>siUSP48	BRCA1, 53BP1	Two-colour analysis	BRCA1	53BP1	0.75	7.5 (53BP1)<br>6 (BRCA1)	40 (53BP1)<br>14 (BRCA1)	5
Investigating RAD51 and 53BP1 accumulations following USP48 loss	siUSP48	RAD51, 53BP1	Two-colour analysis	RAD51	53BP1	0.75	7.5 (53BP1)<br>6 (RAD51)	40 (53BP1)<br>14 (RAD51)	6
Investigating organisation of RAD51, 53BP1 and BRCA1 following USP48 loss	siUSP48	BRCA1, 53BP1, RAD51	Four-colour analysis	RAD51	53BP1<br>BRCA1	0.75	7.5 (53BP1)<br>6 (BRCA1, RAD51)	40 (53BP1)<br>14 (BRCA1, RAD51)	10

[xi] In all experiments, cells were treated with EdU (for cell cycle phase classification) prior to irradiation (2 Gy) and allowed 1 hour to recover.

Table S8. Defining the positioning of BRCA1: 53BP1 following depletion of chromatin regulators, SMARCAD1 and USP48.

Definition of 53BP1 profile	Separation distance of 53BP1 from core BRCA1 (µm)
Control	~1.8-2
Reduced	<0.5
Increased	~2-2.5

Journal of Cell Science • Supplementary information
