# Supplementary material for: Imaging nanoscale nuclear structures with expansion microscopy
Source: J Cell Sci. 2022 Jul 19;135(14):jcs259009. doi: 10.1242/jcs.259009 (PMC9450888; doi:10.1242/jcs.259009)
Supplement: Reviewer comments [file joces-135-259009_review_history.pdf]

Imaging nanoscale nuclear structures with expansion microscopy

Emma L. Faulkner, Jeremy A. Pike, Ruth M. Densham, Evelyn Garlick, Steven G. Thomas, Robert K. Neely and Joanna R. Morris
DOI: 10.1242/jcs.259009

Editor: Jennifer Lippincott-Schwartz

Review timeline

Original submission:	7 June 2021
Editorial decision:	23 August 2021
First revision received:	20 January 2022
Editorial decision:	11 March 2022
Second revision received:	26 May 2022
Accepted:	21 June 2022

Original submission

First decision letter

MS ID#: JOCES/2021/259009

MS TITLE: Imaging Nanoscale Nuclear Structures with Expansion Microscopy

AUTHORS: Robert K Neely, Joanna R Morris, Emma L Faulkner, Steve Thomas, Jeremy A Pike, Ruth M Densham, and Evelyn Garlick
ARTICLE TYPE: Research Article

We have now reached a decision on the above manuscript.

To see the reviewers' reports and a copy of this decision letter, please go to: https://submit-jcs.biologists.org and click on the 'Manuscripts with Decisions' queue in the Author Area. (Corresponding author only has access to reviews.)

As you will see, the reviewers raise a number of substantial criticisms that prevent me from accepting the paper at this stage. They suggest, however, that a revised version might prove acceptable, if you can address their concerns. If you think that you can deal satisfactorily with the criticisms on revision, I would be pleased to see a revised manuscript. We would then return it to the reviewers.

We are aware that you may be experiencing disruption to the normal running of your lab that makes experimental revisions challenging. If it would be helpful, we encourage you to contact us to discuss your revision in greater detail. Please send us a point-by-point response indicating where you are able to address concerns raised (either experimentally or by changes to the text) and where you will not be able to do so within the normal timeframe of a revision. We will then provide further guidance. Please also note that we are happy to extend revision timeframes as necessary.

Please ensure that you clearly highlight all changes made in the revised manuscript. Please avoid using 'Tracked changes' in Word files as these are lost in PDF conversion.

I should be grateful if you would also provide a point-by-point response detailing how you have dealt with the points raised by the reviewers in the 'Response to Reviewers' box. Please attend to

© 2022. Published by The Company of Biologists under the terms of the Creative Commons Attribution License (https://creativecommons.org/licenses/by/4.0/).

all of the reviewers' comments. If you do not agree with any of their criticisms or suggestions please explain clearly why this is so.

Reviewer 1

Advance summary and potential significance to field

Faulkner and colleagues evaluate the application of expansion microscopy for the imaging on nuclear nanostructures by imaging and analyzing nuclear foci formation of key factors of DNA double-strand break repair upon damage induction. The authors are the first to use this innovative, yet accessible, super-resolution imaging approach to assess nuclear DNA-associated protein structures. To validate the approach, the authors confirm observations seen before with regular microscopy or other super-resolution techniques on foci formation of 53BP1 and BRCA1 upon DNA damage. The manuscript ends with interesting observations on the appearance of 53BP1, BRCA1 and RAD51 foci upon damage.
With these last observations, the authors show that super-resolution imaging of DNA damage repair structures holds great promise to better understand how these repair factories function. With their described expansion microscopy, such imaging approaches are accessible to a broad audience, since it only requires standard microscopes available in most institutes. Furthermore, the application of expansion microscopy holds promise for many other research questions studying nuclear protein structures (replication, transcription, etc). Therefore, I feel this manuscript is of great importance to the molecular biology field.

Comments for the author

Major comment:
- The authors have included validation using EdU staining that there is isotropic expansion of the nucleus.
What I miss is validation that expansion microscopy does not yield artefacts in antibody staining. For example, the authors could include a bona fide DSB repair marker (gH2AX) in their stainings to validate that the analyzed structures are at sites of DSBs. Furthermore, the authors could include simple foci quantification to validate similar foci numbers per cell (e.g. 53BP1, gH2AX) using standard confocal imaging pre-expansion and post-expansion.

Minor comment:
- In the figure legends, the authors nicely indicate how many structures were assessed per condition. In some legends, also the number of cells from which the structures originate are indicated. This is important information, especially since the experiments include transient transfections and hence there might be cell-
to-cell variation. It would be good to include the cell numbers for all quantifications (e.g. Fig. 3, Suppl. Fig. 7).

Reviewer 2

Advance summary and potential significance to field

Chromatin structure is dynamically shaped by external stimuli by which cells adapt to the changing environment. It underpins the interaction of transcription factors, histones, and gene regulatory elements to collectively impact transcription. Many different approaches - including sequencing, super-resolution, and electron microscopy methods have been used to better delineate the relationship between structure and function of the chromatin. In Fauklner et al, the authors developed a novel expansion microscopy technique to study the chromatin structure and the changes of the protein distribution upon DNA damage. This provides a potentially high throughput method to understand chromatin structure and its changes in different conditions.

© 2022. Published by The Company of Biologists under the terms of the Creative Commons Attribution License (https://creativecommons.org/licenses/by/4.0/).

Comments for the author

The authors described a novel method to perform isotropic expansion microscopy for the chromatin. This is achieved by anchoring the nucleic acids to the gel.
However, this premise is not well supported in the manuscript. The overlay images of pre-expansion SIM and post-expansion wide field images serve as the only evidence that the expansion is isotropic (Figure 1). This is insufficient. First, it lacks quantification of the co-localization. Second as SIM offers resolution at around 100-130 nm, one cannot argue that the expansion is isotropic at the nanoscale even if the co-localization is perfect. To make a stronger argument, a careful study of the expansion such as Ruixuan et al, 2021 (https://doi.org/10.1038/s41565-021-00875-7) should be done.
The rest of the data is decent. However, it lacks solid foundation for the reasons above regarding Figure 1 and the overarching premise of the manuscript.

Reviewer 3

Advance summary and potential significance to field

The authors present an alternative to traditional superresolution microscopy for investigating the nanoscale organization of DNA-damage response proteins 53BP1 and BRCA1, namely expansion microscopy (ExM).
Through isotropic expansion of the nucleus of human epithelial cells they are able to assess thousands of nanoscale nuclear features, demonstrating how ExM can be applied for studying the nanoscale structure of the nucleus.

Comments for the author

Foundationally, the story presented here is quite nice and makes logical sense. However, there is room for growth before this is ready for publication.
The authors first asses isotropic expansion of the nucleus. To do this they qualitatively compared SIM images of pre-expanded nuclei to widefield images of post-expanded nuclei. I am curious why widefield was used for post-expanded nuclei for this assessment? The imaging quality post-expansion is educed as compared to the pre-expansion SIM images. While there appears to be vague, general agreement it is hard to draw conclusions about “isotropic expansion” from these images (especially in the z-dimension). A slightly more quantitative assessment of expansion was done by measuring the volume of pre- and post-expanded nuclei.
Again, widefield microscopy was used to measure the volume in post-expanded nuclei.
Critique: Before claiming “isotropic” expansion, more analysis should be performed and/or imaging with better axial resolution.
The authors next confirm the organization of two dsDNA break repair regulator proteins, BRCA1 and 53BP1 in S-phase cells following gamma irradiation. They qualitatively assess the localization compared to what is reported in the literature and find good agreement. They then develop a semi-automated sot detection-based analysis to more quantitively measure these protein’s organization. Using this method over thousands of structures in tens of nuclei, 5 different classes of structures were determined. This analysis method is thorough, and the number of structures analyzed is applaudable.
Minor Critique: Wherever possible red/green images should be avoided. They are indistinguishable for many people.
The authors further investigate the distribution of 53BP1 and find that there is a discontinuous spot-like appearance (similar to previously reported STED microscopy), confirm the chromatin-mediated relationship between 53BP1 and BRCA1 (as previous described using confocal microscopy), and observe an increase in RAD51 accumulation following USP48 depletion (in agreement with previous reports).

General Comments:
It is understandable that the purpose of the paper is to describe ExM as an alternative to superresolution microscopy in the nucleus. Therefore, no new biological insight is provided and instead current reporting in the literature are used as a benchmark. What would be useful for the reader is a clearer framework of the

© 2022. Published by The Company of Biologists under the terms of the Creative Commons Attribution License (https://creativecommons.org/licenses/by/4.0/).

“benchmarks” chosen. For instance, what resolution was needed previously vs what can be achieved now? Or how much time does ExM save vs methods previously reported? Making these clear distinctions for the reader will help to show the technological benefit and usefulness of ExM over traditional methods. Without those direct comparisons this work seems redundant.
Similarly, it would be helpful for the reader to clearly know what reportings are “known” (i.e., previously reported) and what is a “new” observation. The discussion starts to clarify this when pointing to “some surprising observations…” but it would be helpful to have this clearly stated in the results as well.

First revision

Author response to reviewers' comments

Reviewer 1 Advance Summary and Potential Significance to Field:
Faulkner and colleagues evaluate the application of expansion microscopy for the imaging on nuclear nanostructures by imaging and analyzing nuclear foci formation of key factors of DNA double-strand break repair upon damage induction. The authors are the first to use this innovative, yet accessible, super-resolution imaging approach to assess nuclear DNA-associated protein structures. To validate the approach, the authors confirm observations seen before with regular microscopy or other super-resolution techniques on foci formation of 53BP1 and BRCA1 upon DNA damage. The manuscript ends with interesting observations on the appearance of 53BP1, BRCA1 and RAD51 foci upon damage.
With these last observations, the authors show that super-resolution imaging of DNA damage repair structures holds great promise to better understand how these repair factories function. With their described expansion microscopy, such imaging approaches are accessible to a broad audience, since it only requires standard microscopes available in most institutes. Furthermore, the application of expansion microscopy holds promise for many other research questions studying nuclear protein structures (replication, transcription, etc). Therefore, I feel this manuscript is of great importance to the molecular biology field.

Reviewer 1 Comments for the Author:

Major comment:

The authors have included validation using EdU staining that there is isotropic expansion of the nucleus. What I miss is validation that expansion microscopy does not yield artefacts in antibody staining. For example, the authors could include a bona fide DSB repair marker (gH2AX) in their staining to validate that the analyzed structures are at sites of DSBs. Furthermore, the authors could include simple foci quantification to validate similar foci numbers per cell (e.g. 53BP1, gH2AX) using standard confocal imaging pre-expansion and post-expansion.

We thank the reviewer for these comments. Our aim indeed, was to test the degree of any potential artefacts (antibody and others) in the modified ExM technique. We have performed foci quantification of BRCA1 and 53BP1 in pre- and post-expansion nuclei as suggested. These data show a comparable distribution of focus numbers pre- and post-expansion (Fig. S1E). We chose the analysis of BRCA1 and 53BP1 in irradiated samples for analysis in this study because they are *bona fide* repair proteins and because BRCA1:53BP1 structures are one of the few repair protein structures whose relationship has been measured previously by SRM using antibodies. This allowed us to assess any potential artefacts in antibody staining that might arise. (Moreover, their relationship with chromatin has been described allowing us to test the degree to which our ExM protocol can, or cannot, report on chromatin-regulated events). Our findings are encouraging and suggest the modified ExM will be useful. However, we accept that the potential for artefact remains and that this needs to be made more explicit in the report. To make the potential for artefact as clear as we can to the reader, we now provide a table detailing the measurements made in previous examination of these structures compared to those in the current study (Table S1).

© 2022. Published by The Company of Biologists under the terms of the Creative Commons Attribution License (https://creativecommons.org/licenses/by/4.0/).

Minor comment:

In the figure legends, the authors nicely indicate how many structures were assessed per condition. In some legends, also the number of cells from which the structures originate are indicated. This is important information, especially since the experiments include transient transfections and hence there might be cell- to-cell variation. It would be good to include the cell numbers for all quantifications (e.g. Fig. 3, Suppl. Fig. 7).

In response to this comment, we have included cell numbers for all quantifications which were absent in the previous submission in Fig. 3, Fig. S3.

Reviewer 2 Advance Summary and Potential Significance to Field:

Chromatin structure is dynamically shaped by external stimuli by which cells adapt to the changing environment. It underpins the interaction of transcription factors, histones, and gene regulatory elements to collectively impact transcription. Many different approaches - including sequencing, super-resolution, and electron microscopy methods have been used to better delineate the relationship between structure and function of the chromatin. In Faulkner et al, the authors developed a novel expansion microscopy technique to study the chromatin structure and the changes of the protein distribution upon DNA damage. This provides a potentially high throughput method to understand chromatin structure and its changes in different conditions.

Reviewer 2 Comments for the Author:

The authors described a novel method to perform isotropic expansion microscopy for the chromatin. This is achieved by anchoring the nucleic acids to the gel. However, this premise is not well supported in the manuscript. The overlay images of pre-expansion SIM and post-expansion wide field images serve as the only evidence that the expansion is isotropic (Figure 1). This is insufficient. First, it lacks quantification of the co-localization. Second, as SIM offers resolution at around 100-130 nm, one cannot argue that the expansion is isotropic at the nanoscale even if the co-localization is perfect. To make a stronger argument, a careful study of the expansion such as Ruixuan et al, 2021 (https://doi.org/10.1038/s41565-021-00875-7) should be done. The rest of the data is decent. However, it lacks solid foundation for the reasons above regarding Figure 1 and the overarching premise of the manuscript.

We thank the reviewer for these comments. We take the point that precise isotropy measurements would be ideal, and since in the context of the nucleus we cannot measure a known, stable structure (such as microtubules or the NPC as referenced in Ruixan *et al*) we have made it clear to the reader the limits of our ability to claim isotropy. Namely that with a pre-expansion SIM resolution of 120-130 nm, we can only discount distortions above this resolution (page 5, line 117-118). Additionally, we provide a table detailing the measurements made in previous examination of 53BP1:BRCA1 structures (at 250-300 nm resolution) compared to those in the current study (Table S1). This illustrates congruency of our findings using modified ExM with those of previous modalities and is consistent with minimal distortion at the resolution of 65-70 nm.

Further we have repeated the correlative imaging experiment (Fig. 1). We performed registration of the pre-expansion SIM and post-expansion widefield imaging using a customised Matlab graphical user interface. In this approach, we manually placed points on features in each of the images and these were used to perform the subsequent registration transform (details of this provided on page 5, lines 112-115 and in the methods (page 18, lines 433-445)) We have then measured the mean squared error (MSE) between pairwise points of interest within the images. Challenges we noted in this approach is that, unlike the features commonly used for validation in ExM (e.g. microtubules), nuclear architecture lacks easily identifiable, discrete features for comparison between each image. The axial expansion factor further compounds this issue and makes judgement of feature placement for registration and measurements difficult. Nonetheless, using a scaled, rigid rotation, we obtained good visual correlation between the pre- and post-ExM images (Fig. 1A-B) and the MSE between control points in the images showed a 1:1 ratio regardless of distance between measurements (Fig.1C). We speculate any error is derived from the difficulties identifying features precisely in 3D as discussed above.

© 2022. Published by The Company of Biologists under the terms of the Creative Commons Attribution License (https://creativecommons.org/licenses/by/4.0/).

Reviewer 3 Advance Summary and Potential Significance to Field:

The authors present an alternative to traditional super resolution microscopy for investigating the nanoscale organization of DNA-damage response proteins 53BP1 and BRCA1, namely expansion microscopy (ExM). Through isotropic expansion of the nucleus of human epithelial cells they are able to assess thousands of nanoscale nuclear features, demonstrating how ExM can be applied for studying the nanoscale structure of the nucleus.

Reviewer 3 Comments for the Author:

Foundationally, the story presented here is quite nice and makes logical sense. However, there is room for growth before this is ready for publication. The authors first asses isotropic expansion of the nucleus. To do this they qualitatively compared SIM images of pre-expanded nuclei to widefield images of post-expanded nuclei. I am curious why widefield was used for post-expanded nuclei for this assessment? The imaging quality post-expansion is reduced as compared to the pre-expansion SIM images.

We thank the reviewer for this feedback. The correlative imaging approach commonly applied to validate isotropic expansion involves acquisition of pre-expansion images using a super resolved system e.g. SIM which can result in fluorophore bleaching, thereby limiting the photon budget available after expansion. This effect is further compounded by the volumetric dilution of fluorescence which is inherent to ExM. To overcome these potential limitations, we opted to use widefield imaging to minimise the irradiating photon density and fluorophore photobleaching. We have since repeated this experiment to obtain better quality images (new Fig. 1).

While there appears to be vague, general agreement it is hard to draw conclusions about “isotropic expansion” from these images (especially in the z-dimension). A slightly more quantitative assessment of expansion was done by measuring the volume of pre- and post-expanded nuclei. Again, widefield microscopy was used to measure the volume in post-expanded nuclei. Critique: Before claiming “isotropic” expansion, more analysis should be performed and/or imaging with better axial resolution.

We agree that due to the four-fold expansion in the z-axis, finding agreement within the images is extremely challenging. Such analyses are traditionally performed with microtubules or NPCs which have a characteristic molecular configuration, and this may aid in matching features within the Z-axis. In this case, the complex topology of the nucleus presents a challenge in clearly identifying features before and after expansion. To address this, we repeated the correlative imaging experiment to obtain better quality images. We visually found good agreement of features between the pre-expansion SIM and post-expansion widefield images (Fig. 1A-B). We performed image registration by selecting points of interest in both images and using these as a basis for the transformation (details of this provided on page 5, lines 112-115 and in the methods (page 18, lines 433-445)). The mean squared error (MSE) was determined between pairwise points in the images, producing 1:1 ratio between pre- and post-ExM measurements (Fig. 1C). We speculate any error is derived from the difficulties identifying features precisely in 3D as discussed above.

The authors next confirm the organization of two dsDNA break repair regulator proteins, BRCA1 and 53BP1 in S-phase cells following gamma irradiation. They qualitatively assess the localization compared to what is reported in the literature and find good agreement. They then develop a semi-automated sot detection-based analysis to more quantitatively measure these protein’s organization. Using this method over thousands of structures in tens of nuclei, 5 different classes of structures were determined. This analysis method is thorough, and the number of structures analyzed is applaudable.
Minor Critique: Wherever possible red/green images should be avoided. They are indistinguishable for many people.

Look up tables in images have been altered throughout the manuscript, replacing red/ green for magenta/ green images.

The authors further investigate the distribution of 53BP1 and find that there is a discontinuous spot-like appearance (similar to previously reported STED microscopy), confirm the chromatin-

© 2022. Published by The Company of Biologists under the terms of the Creative Commons Attribution License (https://creativecommons.org/licenses/by/4.0/).

mediated relationship between 53BP1 and BRCA1 (as previous described using confocal microscopy), and observe an increase in RAD51 accumulation following USP48 depletion (in agreement with previous reports).

General Comments:

It is understandable that the purpose of the paper is to describe ExM as an alternative to super-resolution microscopy in the nucleus. Therefore, no new biological insight is provided and instead current reporting in the literature are used as a benchmark. What would be useful for the reader is a clearer framework of the “benchmarks” chosen. For instance, what resolution was needed previously vs what can be achieved now? Or how much time does ExM save vs methods previously reported? Making these clear distinctions for the reader will help to show the technological benefit and usefulness of ExM over traditional methods. Without those direct comparisons this work seems redundant.

We now provide a table detailing the measurements made in previous examination of these structures compared to those in the current study, so that the benchmarks are clear (Table S1). Further to clarify the advantages of ExM, we have rephrased the paragraph in the discussion section (page 9, lines 223-225 and page 10 lines 237-248), outlining where ExM overcomes limitations of traditional super-resolution techniques. The main advantages we highlight are an ability to acquire 3D nanoscale images of features rapidly as images are acquired on a conventional diffraction-limited system; and that imaging volume and depth are improved compared to traditional methods. These features make it possible to acquire thousands of features (compared to 10s-100s with other SRM techniques) which can be quantitatively described at the nanoscale using a spot-detection based analysis.

Similarly, it would be helpful for the reader to clearly know what reportings are “known” (i.e., previously reported) and what is a “new” observation. The discussion starts to clarify this when pointing to “some surprising observations…” but it would be helpful to have this clearly stated in the results as well.

To clarify which observations are known or new, we have added a comment to the final paragraph in Nanoscale organisation of DNA-damage signalling proteins section (page 6, lines 133-137). We have also restructured the Averaging hundreds of nanoscale features to explore chromatin regulators to make clear where we are confirming previous observations on effects observed by confocal microscopy (page 7, lines 170-172 and page 7 lines 178-181). We also provide a table summarising our observations compared to previous studies (Table S1).

Further changes to the manuscript

Main text figure changes:

LUTs and schematics changed from red to magenta for 53BP1 channel throughout.
Fig. 2: Merged Fig. S3 and S6 from original submission with Fig. 2 in the main text.

Supplementary Figure changes:

Fig. S2 removed.

Fig. S3 merged with Fig. 2 now Fig. 2B-C

Fig. S4 renumbered as Fig. S2

Fig. S5 renumbered as Fig. S3. Schematic of classes 1-5 altered and now generically shows spatial organisation of 53BP1 and BRCA1 as part A (part C removed). Fig. S7C&D merged with this figure, now labelled Fig. S3D-E.

Fig. S6 merged with Fig. 2 now Fig. 2D.

© 2022. Published by The Company of Biologists under the terms of the Creative Commons Attribution License (https://creativecommons.org/licenses/by/4.0/).

Fig. S7 orthogonal views of class 5 structures following SMARCAD1/ USP48 depletion (Fig. S7A-B) removed and commented upon in manuscript (data not shown). Fig. S7C-D merged with Fig. S3 now numbered Fig. S3D-E.

Fig. S8 A-B now numbered Fig. S4A-B. Fig. S8C-D removed and included as a table.

Fig. S9A-C merged with Fig. S4 now numbered Fig. S4C-E.

Fig. S10 removed and included as a table.

Second decision letter

MS ID#: JOCES/2021/259009

MS TITLE: Imaging Nanoscale Nuclear Structures with Expansion Microscopy

AUTHORS: Emma L Faulkner, Jeremy A Pike, Ruth M Densham, Evelyn Garlick, Steve Thomas, Robert K Neely, and Joanna R Morris
ARTICLE TYPE: Research Article

We have now reached a decision on the above manuscript.

To see the reviewers' reports and a copy of this decision letter, please go to: https://submit-jcs.biologists.organd click on the 'Manuscripts with Decisions' queue in the Author Area. (Corresponding author only has access to reviews.)

As you will see, the reviewers gave favourable reports but reviewer #2 raised some critical points that will require amendments to your manuscript. Specifically, can you please address reviewer #2's concern about clarifying the number of samples you have analyzed using the same correlative method and whether it is highly reproducible across samples prepared at separate times? I hope that you will be able to carry these out because I would like to be able to accept your paper, depending on further comments from reviewers.

We are aware that you may be experiencing disruption to the normal running of your lab that makes experimental revisions challenging. If it would be helpful, we encourage you to contact us to discuss your revision in greater detail. Please send us a point-by-point response indicating where you are able to address concerns raised (either experimentally or by changes to the text) and where you will not be able to do so within the normal timeframe of a revision. We will then provide further guidance. Please also note that we are happy to extend revision timeframes as necessary.

Please ensure that you clearly highlight all changes made in the revised manuscript. Please avoid using 'Tracked changes' in Word files as these are lost in PDF conversion.

I should be grateful if you would also provide a point-by-point response detailing how you have dealt with the points raised by the reviewers in the 'Response to Reviewers' box. Please attend to all of the reviewers' comments. If you do not agree with any of their criticisms or suggestions please explain clearly why this is so.

Reviewer 1

Advance summary and potential significance to field

In this revised manuscript, the authors have reacted to my comments in an appropriate way. The extra validation is an important addition to prove the minimal potential of artefacts of ExM.
Comments for the author

N/A

© 2022. Published by The Company of Biologists under the terms of the Creative Commons Attribution License (https://creativecommons.org/licenses/by/4.0/).

Reviewer 2

Advance summary and potential significance to field

Faulkner et al. described a novel method to perform expansion microscopy for the nuclear compartment and applied the technique to study the spatial relationship for several DNA damage response proteins. This methodology can greatly facilitate many future studies that aim to address structure and function of the chromatin. It can also as the foundation for future improvements on expansion microscopy for the nucleus.

Comments for the author

The data that supports the isotropic expansion of the nucleus is now much more compelling (Figure 1), which anchors the rest of the paper. Can the authors clarify the number of samples they have analyzed using the same correlative method and if it is highly reproducible across samples prepared at separate times? As this method would be of great interest to many people studying the nucleus, it would be helpful to address the reproducibility.
The authors pointed out that the nucleus lacks well-defined landmarks. Some structures that might be worth considering are the nuclear pore complexes and sequence-specific labeling through dCas9-GFP. These could serve as useful labels to determine the correlation pre and post expansion. For the current manuscript especially considering the applications that the authors demonstrated, these experiments are not essential. However, for further method development - for example, registration down to 50-80 nm scale (STED or others), these experiments can be considered.
One minor comment for the abstract. The authors can make further clarification on the isotropic expansion at the nanoscale by providing the quantification mentioned in the Results section, i.e. equal or better than 100 nm.

Second revision

Author response to reviewers' comments

Reviewer 2 Advance Summary and Potential Significance to Field:

Faulkner et al. described a novel method to perform expansion microscopy for the nuclear compartment and applied the technique to study the spatial relationship for several DNA damage response proteins. This methodology can greatly facilitate many future studies that aim to address structure and function of the chromatin. It can also as the foundation for future improvements on expansion microscopy for the nucleus.

Reviewer 2 Comments for the Author:

The data that supports the isotropic expansion of the nucleus is now much more compelling (Figure 1), which anchors the rest of the paper. Can the authors clarify the number of samples they have analysed using the same correlative method and if it is highly reproducible across samples prepared at separate times? As this method would be of great interest to many people studying the nucleus, it would be helpful to address the reproducibility.

We thank the reviewer for these comments. We have performed the correlative imaging experiment on two different biological replicates; the first correlative imaging experiment was provided in our original submission (shown in Figure 1 below). Within these images, we successfully identified the same nuclei and show that their morphology and features are retained post-expansion. We noted due to the presence of the grid lines in the post-expansion widefield images and some artefacts in the reconstructed SIM data. Therefore, these images cannot be analysed using the method described in the present manuscript. Nevertheless, these images support the reproducibility of the expansion of the nuclear compartment. We have clarified that this is reproducible in the results section.

© 2022. Published by The Company of Biologists under the terms of the Creative Commons Attribution License (https://creativecommons.org/licenses/by/4.0/).

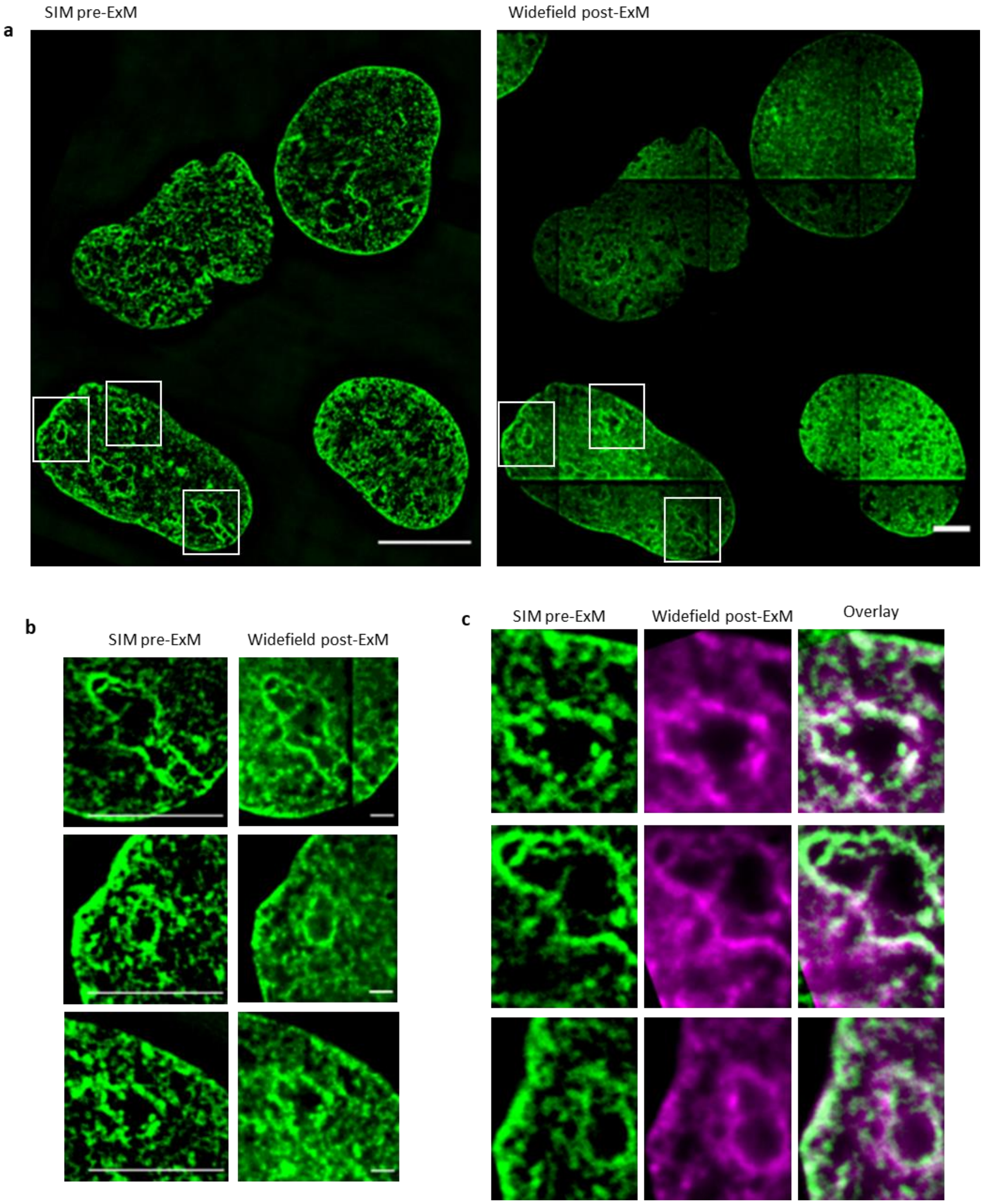

Figure 1. Isotropic expansion of the nucleus. U2OS cells were treated with EdU overnight before fixation. Click it reaction was performed to detect EdU, and images were acquired either pre-ExM or post-ExM.

a) Correlative imaging was performed by acquiring pre-ExM images on a structured illumination microscope in 3D-SIM mode and then acquiring images of the same nuclei post-ExM on a widefield microscope. Scale bars 10 µm (equivalent to ~2.5 µm pre-ExM).
b) Features were selected from the pre- and post-ExM correlative images and compared.
c) Corresponding features in the pre-ExM SIM (green) and post-ExM widefield (magenta) images were identified by eye and registered. Overlays of these features are shown.

© 2022. Published by The Company of Biologists under the terms of the Creative Commons Attribution License (https://creativecommons.org/licenses/by/4.0/).

Additionally, we analysed the arrangement of 53BP1:BRCA1 co-enriched foci in expanded samples. The average structures shown in Figure 3 are representative of hundreds of features from three biological replicates. To test reproducibility across these samples (prepared at different times) we investigated the spatial distribution of 53BP1 and BRCA1 in each biological replicate. To do this, we averaged all class five structures from each biological replicate as shown in Figure 2. We observe consistent organisation of 53BP1:BRCA1 in the structure generated from each replicate.

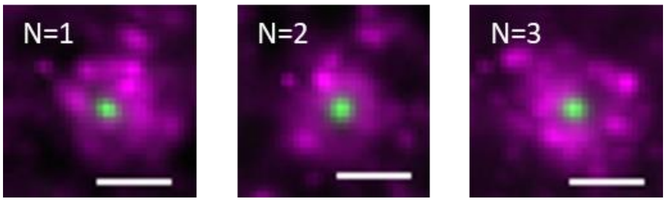

Figure 2. Positioning of BRCA1: 53BP1 following damage induction by irradiation. Average profiles of class five structures (defined as discontinuous 53BP1 spots encapsulating a central BRCA1 spot) from each biological replicate are shown. Examples shown are NTC cells treated as in Figure 3. Scale bars 2 µm. N=1 147 structures, N=2, 237 structures, N=3, 243 structures.

In summary, we have performed correlative imaging experiments on the nuclear architecture in two biological replicates. In both repeats, we successfully identified the same nuclei and observed their morphology and structural organisation was retained post-expansion. Additionally, we investigated the organisation of 53BP1:BRCA1 co-enriched structures across three biological replicates and observed consistent spatial organisation of these features. We therefore conclude that our approach to ExM in the nuclear compartment is highly reproducible.

The authors pointed out that the nucleus lacks well-defined landmarks. Some structures that might be worth considering are the nuclear pore complexes and sequence-specific labeling through dCas9-GFP. These could serve as useful labels to determine the correlation pre and post expansion. For the current manuscript, especially considering the applications that the authors demonstrated, these experiments are not essential. However, for further method development – for example, registration down to 50-80 nm scale (STED or others), these experiments can be considered.

We thank the reviewer for this comment and agree the use of such landmarks would aid in determining the correlation between pre- and post-expansion. We would therefore perform these experiments in future method development.

One minor comment for the abstract. The authors can make further clarification on the isotropic expansion at the nanoscale by providing the quantification mentioned in the Results section, i.e. equal or better than 100 nm.

We have added this clarification to the abstract, stating that we demonstrate isotropic expansion of the nucleus at a resolution equal to or better than 120-130 nm.

Third decision letter

MS ID#: JOCES/2021/259009

MS TITLE: Imaging Nanoscale Nuclear Structures with Expansion Microscopy

AUTHORS: Emma L Faulkner, Jeremy A Pike, Ruth M Densham, Evelyn Garlick, Steve Thomas, Robert K Neely, and Joanna R Morris
ARTICLE TYPE: Research Article

I am happy to tell you that your manuscript has been accepted for publication in Journal of Cell Science, pending standard ethics checks.

© 2022. Published by The Company of Biologists under the terms of the Creative Commons Attribution License (https://creativecommons.org/licenses/by/4.0/).
